# Next‐generation spatial transcriptomics: unleashing the power to gear up translational oncology

**DOI:** 10.1002/mco2.765

**Published:** 2024-10-06

**Authors:** Nan Wang, Weifeng Hong, Yixing Wu, Zhe‐Sheng Chen, Minghua Bai, Weixin Wang, Ji Zhu

**Affiliations:** ^1^ Cosmos Wisdom Biotech Co. Ltd Hangzhou China; ^2^ Department of Radiation Oncology Zhejiang Cancer Hospital Hangzhou China; ^3^ Hangzhou Institute of Medicine (HIM) Chinese Academy of Sciences Hangzhou China; ^4^ Zhejiang Key Laboratory of Radiation Oncology Hangzhou China; ^5^ Department of Pulmonary and Critical Care Medicine Zhongshan Hospital Fudan University Shanghai China; ^6^ Department of Pharmaceutical Sciences College of Pharmacy and Health Sciences Institute for Biotechnology St. John's University Queens New York USA

**Keywords:** biomarker profiling, mechanism elucidation, oncology, single‐cell resolution, spatial transcriptomics, tumor microenvironment

## Abstract

The growing advances in spatial transcriptomics (ST) stand as the new frontier bringing unprecedented influences in the realm of translational oncology. This has triggered systemic experimental design, analytical scope, and depth alongside with thorough bioinformatics approaches being constantly developed in the last few years. However, harnessing the power of spatial biology and streamlining an array of ST tools to achieve designated research goals are fundamental and require real‐world experiences. We present a systemic review by updating the technical scope of ST across different principal basis in a timeline manner hinting on the generally adopted ST techniques used within the community. We also review the current progress of bioinformatic tools and propose in a pipelined workflow with a toolbox available for ST data exploration. With particular interests in tumor microenvironment where ST is being broadly utilized, we summarize the up‐to‐date progress made via ST‐based technologies by narrating studies categorized into either mechanistic elucidation or biomarker profiling (translational oncology) across multiple cancer types and their ways of deploying the research through ST. This updated review offers as a guidance with forward‐looking viewpoints endorsed by many high‐resolution ST tools being utilized to disentangle biological questions that may lead to clinical significance in the future.

## Graphical abstract

Proposed biomarker development pipeline using spatial transcriptome. Schematic illustration of biomarker profiling workflow based on ST. Cancer types are listed on the left for demonstrative purposes (see abbreviations). Sampling strategies are divided into full tissue sections and tissue microarrays (TMA) depending on ST technologies as exploratory tools (ISS/ISH‐ST, array‐based ST, and ROI‐ST). Bioinformatic explorations (listed in boxes) are grouped accordingly to different ST technologies being applied. Downstream validation is for demonstration but involves increasing number of samples and decreasing number of targets. *Abbreviations*: BRCA, breast carcinoma; COAD, colon adenocarcinoma; ESSC, esophageal carcinoma; GBM, glioblastoma multiforme; HNSC, head and neck squamous cell carcinoma; KIRC, kidney renal cancer; LUAD, lung adenocarcinoma; PDAC, pancreatic ductal carcinoma; DLBC, lymphoid diffuse large B‐cell lymphoma; HCC, hepatocellular carcinoma.

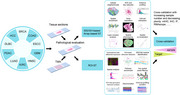

## 1 INTRODUCTION

Influenced by the wide‐spread application of single‐cell transcriptomics (mainly via single‐cell RNA sequence, scRNA‐seq), understanding the cellular dynamics within the complex tissue microenvironment is not limited to decipher each cell's identity via its “omics” profiles but goes further to put them under a histological context. In various research settings—including organ development, embryonic morphogenesis, cognitive science (such as brain theory and neuroscience), and pathologies (including infection‐related pathogenesis, neurodegenerative diseases, and oncogenesis)—the three‐dimensional (3D) interplay of cells dictates biological functions.

In tissues, multiple cellular niches play significant roles such as intercellular modulation via direct contact or short‐ranged paracrine that reprograms biological processes or chemical/molecular dispersion under organ‐specific conditions in a patterned manner. As a result, the spatially compiled multiparameter data with high resolution become useful resources to open up a new field in many biology settings and same as the case in tumor biology. Since the application of ST grew exponentially in the past 3 years and true single‐cell ST methods were gradually becoming available, to update their progress, adaptation of bioinformatics tools in real practice and application frontiers in tumor biology, we provide this systemic review.

Here, we propose the rationale of applying spatial transcriptomics (ST), the building blocks of spatial biology for hypothesis‐free discovery and summarize the historical development path of various ST technologies on their relevant ground basis. With particular interests in tissue biology including cancer, we review the major bioinformatics tools being adopted in the past few years and present an analytical schema compatible with different ST approaches. We also highlight major ST works contributing toward tumor biology and translational oncology wherein spatial single‐cell resolved ST is specifically emphasized. These altogether shed light on future direction and application of ST on a multilayered perspective.

## 2 THE APPLICATION RATIONALE OF ST IN TUMOR BIOLOGY

**FIGURE 1:**
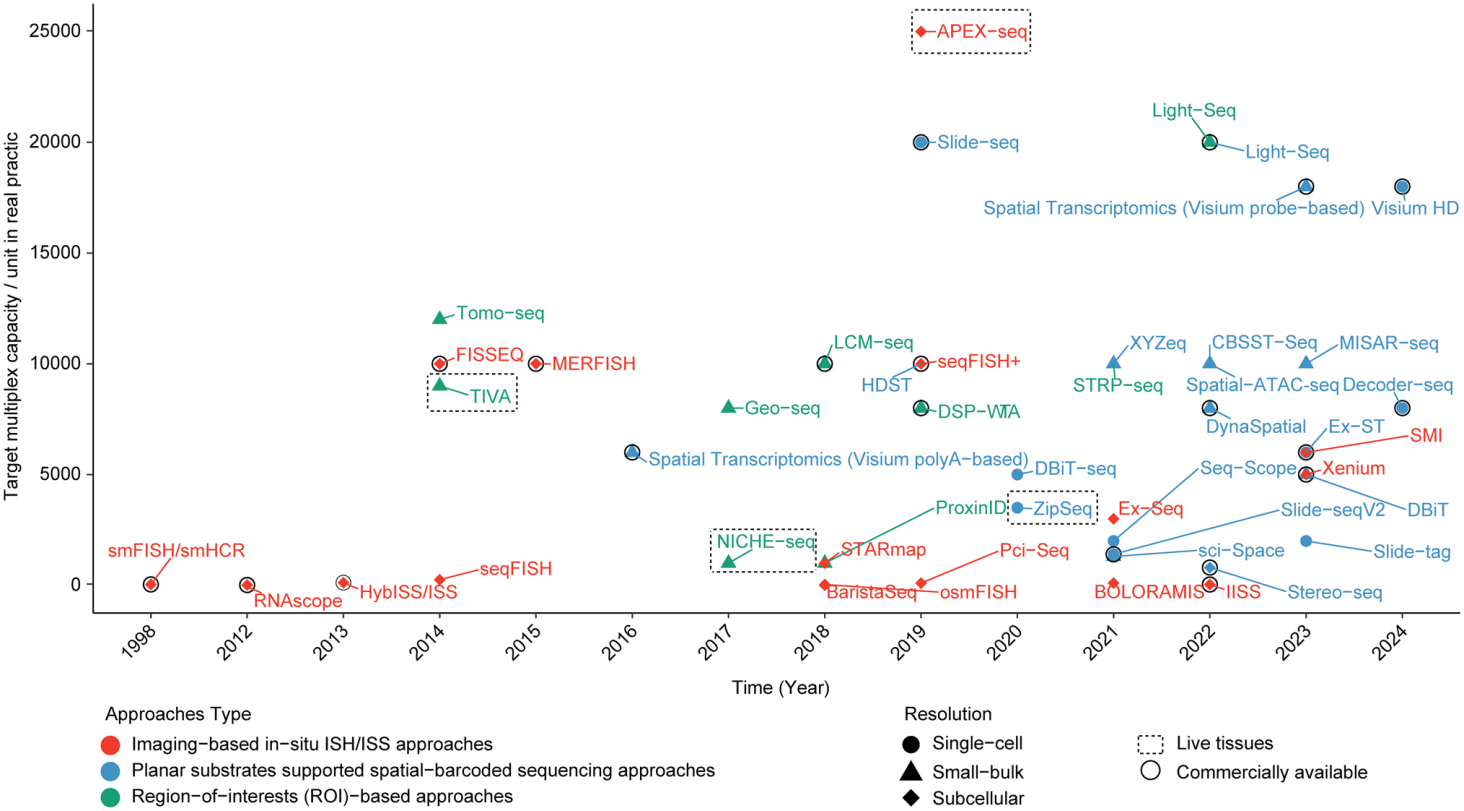
The development roadmap of spatial transcriptomics (ST). Individual ST technologies are presented in a timeline graph between 1998 and 2024 (current). Colors annotate different categories based on their analytical principles including imaging‐based in situ ISH/ISS (red) and planar substrates supported spatial‐barcoded sequencing (blue). From application perspective, other regions‐of‐interest (ROI) based technologies (light‐guided and image‐free) are grouped in green color. Spatial resolutions are presented in different shapes (triangle: small bulk, circle: single‐cell, diamond: subcellular). Black circles indicate technologies that are commercially available. Dashed rectangles indicate application scenario (live tissues). Horizontal axis shows the multiplexity of technologies. *Note*: data summarized may not include all relevant technologies and target plexity is a rough estimate based on references.

Tissues, composed of millions of cells and other cellular functional units, are indispensable resources to gain biological insights under various pathophysiological settings. In tumor biology, pathological evaluation offers as a gold‐standard approach throughout decades and therefore becomes the “central dogma” for clinical decision making.^1^ In‐depth characterization of tissues essentially by compiling multilayers of molecular information now goes much beyond conventional genetics/epigenetics, transcriptomics, proteomics, and metabolomics treating tissues as a whole but rather can be conducted in a decomposed manner at subhistological or individual cell dimensions. Through this magnifying lens, multiomics profiling can be revisited and potentially redefine our comprehension toward disease conditions making novel breakthroughs in translational oncology. Prior to this, scRNA‐seq together with other single‐cell omics (including multiplex single‐cell proteomics and single‐cell analysis of accessible chromatin) has already pioneered in this field but rapid development and maturation of ST further enables in situ profiling of dynamic changes at the transcriptome‐wide scale highlighting the prerequisite of adding locational information in exploring histological specimens.^2^ In the following section, we give a holistic overview of various ST technologies categorized by their base principles and technical strength with a particular emphasis on those that are already available in general research communities. The original technical papers and review summaries are referable in related publications and will not be elaborated here.^2^, ^3^, ^4^, ^5^, ^6^, ^7^, ^8^, ^9^ (Figure 1)

## 3 THE ONCOMING ERA OF TECHNICAL AND ANALYTICAL DEVELOPMENT IN ST

### 3.1 Upstream technology development and current progress

The development paths of various ST can generally be traced back according to their detection principles, which either reply upon conventional next‐generation sequencing (NGS), or in situ hybridization (ISH)/in situ sequencing (ISS), the latter of which share similar principle in signal readouts but differ in their intermediate procedures. From the application aspect, since a subset of technologies requires laser‐captured microdissection (LCM) such as geographical position sequencing (Geo‐seq) and ProximID^10^, ^11^ or photocleavable linkers to extract part of tissue materials/information to generate NGS‐based transcriptomic profiles (transcriptome in vivo analysis: TIVA, NICHE‐seq, Light‐seq, and GeoMx digital spatial profiler whole transcriptome analysis: DSP‐WTA)^12^, ^13^, ^14^, ^15^ or rather employs image‐free spatial information reconstruction approaches (tomo‐seq and STRP‐seq) that are often used under specific settings, we group these technologies in a category termed region‐of‐interests (ROI)‐based spatial approach.^6^, ^16^, ^17^

Though a common feature of those technologies is their limited resolution at a subhistological level that often requires at least a few hundred of cells for downstream profiling, due to their high‐plex potential capable of covering the entire transcriptome, some (such as commercialized GeoMx DSP, nanoString, WA) have already been widely adopted and will be discussed in the below section.

#### 3.1.1 A history of imaging‐based ST technologies

Historically, imaging‐based ST has long track histories and can date back when single RNA molecules could be detected and visualized using conventional fluorescent in situ hybridization (FISH) or single‐molecule FISH (smFISH) at single‐cell resolution and was more recently developed by Nilsson's laboratory using padlock probe‐mediated transcript hybridization followed by rolling‐circle amplification (RCA) for signal amplification and visualization.^18^, ^19^, ^20^ This was subsequently combined with cyclic fluorescence and imaging, a process called in situ serial decoding to allow multiplex target detection.^20^ To facilitate unbiased RNA characterization, Church's laboratory applied di‐nucleotide‐specific fluorescent oligonucleotides to generate a codebook that directly ligates with adapter primers targeting the paired anchoring sequences predesigned within the RCA product complex (ISS‐based).^21^, ^22^ This technique, referred to as fluorescence in situ sequencing (FISSEQ), was initially designed to detect RNA molecules in an un‐targeted way and later, this RCA process was more frequently used as an efficient strategy for in situ signal amplification with or without introducing target‐specific padlock oligonucleotide structures to detect localized RNA transcripts in a targeted manner. Owing to the superior signal‐to‐noise ratio, the RCA‐mediated in situ amplification technologies have vastly revolutionized in the past decade with similar methods being developed such as HybISS/ISS, STARmap, BaristaSeq, Ex‐Seq, and BOLORAMIS with multiplexity ranging from 30 to over 10,000.^21^, ^23^, ^24^, ^25^, ^26^ Recently, the technical advancement also made the commercial form available, which is now being used to address biological questions under various settings including cancer (Xenium in situ; 10X Genomics, CA).^27^, ^28^, ^29^

An alternatively imaging approach to increase detection sensitivity was also introduced following the invention of a novel RNA ISH method: RNA Scope that utilizes paired target RNA‐specific oligonucleotide probes (18‐base each to form a double Z structure) as the docking point to allow overhanging preamplifer oligonucleotides to bind and the free‐floating overhangs contain multiple fluorescence‐oligo hybridization sites allowing signal amplification for visualization.^30^ Similarly, to resolve high‐plex gene profiling, cyclic hybridization is also necessary for producing fluorescence‐based sequential codes. Using this concept, higher plexity can be reached with different analytical scopes and those are exemplified by low target throughput (less than 100) techniques such as smHCR, osmFISH, median throughput methods (100–1,000) such as sequential FISH (seqFISH), and high‐throughput techniques (over 10,000) such as seqFISH+ (an upgrade version of seqFISH) and multiplexed error‐robust FISH (MERFISH).^6^, ^8^, ^31^, ^32^, ^33^, ^34^, ^35^ Theoretically, by applying multiple target‐specific fluorescence‐conjugated DNA probes in each hybridization round, signals can be successfully detected and quantitatively resolved using specific decoding strategies. In brief, the latest high‐throughput versions such as seqFISH+ and MERFISH use target‐specific saddle probe sets as landing points to capture fluorescence‐labeled readout probes generating exquisitely designed coding schemas via binary 69‐bit harming distance 4 or combinatory pseudocolor readout sequences.^8^, ^34^, ^36^ With super‐resolved imaging systems, tens of thousands of transcripts can be successfully discerned and quantified and these prototypes are now invented commercially as MERSCOPE (Vizgen, MA) and seqFISH (Spatial Genomics, CA), respectively. Other successful counterparts with various detection capacities such as spatial molecular imager (SMI CosMX, nanoString, WA), Molecular Cartography (Resolve Biosciences, GmbH), Rebus Esper (Rebus Biosystems, CA), and SEERNA ISS (Dynamic Biosystems, Suzhou) also started to appear at the application frontier.^2^, ^37^, ^38^, ^39^ A very important application advantage shared across these imaging‐based ST platforms is their capability typically reaching at single cell or even subcellular resolution with analytes (tissues or cells) being unaffected, an approach amenable for parallel pathology examination and downstream multiplex staining.

#### 3.1.2 Spatial barcode‐based ST technologies

Due to the broader application of NGS, recently, spatial barcoding‐based technologies also open a new avenue in the spatial biology field and in particular this was predominantly boosted by the development and application of ST developed by Lundeberg's laboratory, a technology later transformed into its commercial version as Visium ST (10X Genomics).^40^ Essentially, the earlier versions of ST and many other successors label planarly indexed *x*–*y* units or use single‐cell label indentation (spot arrays or microbeads, DNA nanoballs, microfluidic chambers, or other microscale molecules) with predefined oligo sequences per *x*–*y*/analytical unit to create spatial oligo barcodes allowing transcript profiles to project into designated space.^3^, ^41^, ^42^, ^43^, ^44^, ^45^, ^46^, ^47^ In fact, many of these STs employ oligo (dT) primers aligned with unique molecular identifier together with spatial barcode oligos and use the classic polyA capturing method for gene identification and quantification at per spatial coordinate. Since these ST techniques require polyA capture, fresh frozen (FF) samples are primarily used. More recently, to extend the analytical scope and sample compatibility, probe‐based STs were developed such as the upgraded versions of Visium ST including Visium V2 and Visium HD (10X Genomics). Apart from their downstream workflow being nearly identical, the major technical difference lies in its upstream design of genes‐specific DNA oligos that hybridize to complementary RNA in situ and flanking ployA sequence for probe capture followed by library construction and sequencing. Having such an advantage, designing transcriptome‐wide probe sets are becoming feasible.

For ST, despite the analytical resolution spanning from 0.5 to 100 µm per spatial unit, the major challenge remains as to leverage the spatial resolution and transcripts being detected. The major advantage of those technologies is that they do not require extra instrumentation and can be standardized in a typical bench‐side workflow. Therefore, many have been commercialized and wide‐spread across research areas such as neuroscience, development biology and disease pathogenesis including cancer.^48^, ^49^, ^50^, ^51^ Some technologies translating into commercial products include Visium/Visium HD based on ST and HDST (10X Genomics),^40^ Curio Seeker based on Slide‐seq (Curio Bioscience, CA),^42^, ^52^ STOmics based on Stereo‐seq (BGI, Shenzhen),^47^ DBiT‐seq based on DBiT (AtlasXomics, CT),^46^ Dynaspatial based on Decoder‐seq (Dynamic Biosystems),^53^ and BMKMANU S1000 (BMKgene, Qingdao).^54^ Technology‐wise, since most of these technologies are based on direct sequencing of reverse‐transcribed (RT) oligo products, they are compatible for exploratory studies in an unbiased manner and as for resolution, a bunch of them have already reached near single‐cell or subcellular level with multiplexity roughly from few hundred to over 10,000.^2^, ^6^, ^41^, ^55^ More recently, head‐to‐head comparison was conducted aligning 11 sequencing‐based ST methods to assess their transcript capture efficiency, feature gene detection sensitivity and molecular diffusion.^56^ Within those, considering the resolution as the major deterministic factor, Stereo‐seq, Slide‐seq V2, Visium V2, and DynaSpatial outperformed others with regard to their capturing efficiency.^56^

#### 3.1.3 ROI‐based ST technologies

ROI‐based ST often requires NGS sequencing as data output and was primarily introduced when specific tissue regions could be collected using LCM in low plex manner (few targets detected via polymerase chain reaction PCR).^57^ Comprehensive coverage of transcriptome was later achieved and some were compatible with single‐cell resolution such as LCM‐seq, Geo‐seq, and ProximID.^10^, ^11^, ^58^ Instead of sectioning tissues, the alternative approach to detect transcripts with in predefined regions was to employ light‐assisted methods to select ROI. Such include NICHE‐seq that uses photoactivatable fluorescent proteins to allow visual inspection and selection of tissue regions^13^ and others such as Light‐seq, which utilizes light‐directed spatial barcode indentation through photo‐crosslinking at target tissue regions being analyzed via ex situ NGS.^15^ Recently, digital spatial profiling (DSP) was developed and broadly propagated within the community and this technique employs photocleavable linkers and micrometer‐sized digital mirror device for ROI selection coupled with downstream NGS profiling. Due to its probe‐based detection theory via ISH, it is widely adaptable in formalin fixed paraffin‐embedded (FFPE) samples and more advantageously compatible with RNA–protein coprofiling.^14^ Though limited by their spatial resolution typically ranging from 10 to hundreds of cells to start with, these STs are mainly pathologically informed, an important preanalytical factor to be considered.

### 3.2 Application consideration using different ST approaches

Overall, these ROI‐based ST together with aforementioned ISS/ISH and spatial‐barcoded ST have been gradually reshaping our understanding toward tissue molecular biology. In real practice, as for spatial‐barcoded ST, since they are kit‐based and often do not rely on heavy instrumentation, a few of them have been rapidly propagated and used across research fields (Figure 1). Also noted is that although ST technologies developed through different routes are versatile, imaging‐based ST having the longest track‐history, remains as a generally adopted ST approach and even in the high‐plex ST application field, some are already proven to be robust and becoming technically feasible (Figure 1). Another key advantage of image ST is within its wide compatibility for clinically archived FFPE samples, an analytical hinderance for FF samples. Nowadays, even some of spatial barcoded ST are emerging to be FFPE‐compatible (Visium probe‐based and Visium HD)^59^ image ST is still the mainstay for FFPE‐based application. However, recent commercialization of spatial‐barcoded ST (probe‐based) may accelerate in short time. Of note, despite their spatial resolution, most probe‐based ST (mainly include image‐based and some ROI‐based ST) are suited for human and mouse exclusively, a species‐limiting factor to be taken into account, especially the ISS/ISH‐based methods that often employ probe‐based detection. For these single‐cell or even subcellularly resolved ST, comparative analysis was already conducted. These include head‐to‐head comparison between Xenium in situ, Merscope and SMI on FFPE samples and additional Molecular Cartography and HiPlex RNA Scope on a set of FF samples.^60^, ^61^ Despite data being preliminary, considering detection sensitivity (readout detectability), false discovery dates (noise control) and cell type imputation being the key analytical parameters, Merscope and Xenium in situ under many tested scenarios may have more potentials for broader application and have been gradually manifested by others.^62^

Since median gene detection capability at per spatial unit is a major concern in ST, this has been the battlefield for many benchmarking studies. In real‐world practice, one technical challenge is to retain gene features to be detected when increasing analytical resolution (from few hundred micrometers down to 0.5–10 µm in size). Based on the whole transcriptome analysis covering over 18,000 genes, median gene features per spatially indexed unit can typically vary from 8,000 to 10,000 genes per analytical units for ROI‐based ST (in case of DSP with ROI diameters of 50–500 µm), 1000–3000 genes for spatial‐barcoded ST (in case of Visium polyA‐based with spot size of 55 µm) and 10 to a few hundred for spatial‐barcoded ST (in case of Stereo‐seq and Visium HD with 0.5–2 µm per bin) in resolution. Technically, a word of warning is that at near single‐cell resolution, challenges still remain as to characterize over 1000 genes per analytical unit, a consensus quality control (QC) measure widely adopted in scRNA‐seq nowadays. To fill these gaps, a body of computational tools have been developed and will be discussed in below sections.

## 4 HARNESSING THE EVOLVING BIOINFORMATIC TOOLBOX GROWN OUT FROM ST

### 4.1 A brief walkthrough of analysis in ST

The multifaceted technical progress in ST makes bioinformatic tools demanding and is being actively developed. Generally, for ST technologies that usually incorporate thousands of indexed spatial units (primarily led spatial‐barcoded ST such as Visium polyA and Stereo‐seq), a typical spatial data analysis procedure may involve raw data processing/QC, sample integration and normalization across genes/samples, dimension reduction/clustering, and cell annotation. On this basis, exploratory work can be carried out including spatial variable gene (SVG) identification, data deconvolution/mapping, gene expression imputation, cell–cell/gene–gene interaction, dynamic analysis (trajectory/RNA velocity), and other complex spatial analysis such as highly ordered cellular neighborhoods (CN), spatial context profiling, or spatial copy number inferring (applicable mainly on ST with single‐cell resolution).^2^, ^4^, ^63^, ^64^, ^65^, ^66^, ^67^, ^68^ Since the data format derived from ROI‐based ST contain sufficient reads per gene in individual ROIs that mimics large‐scale expression data, they apply another route following conventional high‐throughput analysis used in RNA‐seq incorporating methods such as dimension reduction/clustering, variable gene identification, and pathway enrichment (usually based on Limma or DEseq2, GO and KEGG), unbiased expression module identification (GSEA/GSVA, weighted correlation network analysis WGCNA or other machine learning approaches). Another commonly used approach in ROI‐based ST is spatial deconvolution and many methods have been used such as conventional ssGSEA developed for bulk RNA‐seq analysis.

### 4.2 The toolbox and application of bioinformatics in ST

**FIGURE 2:**
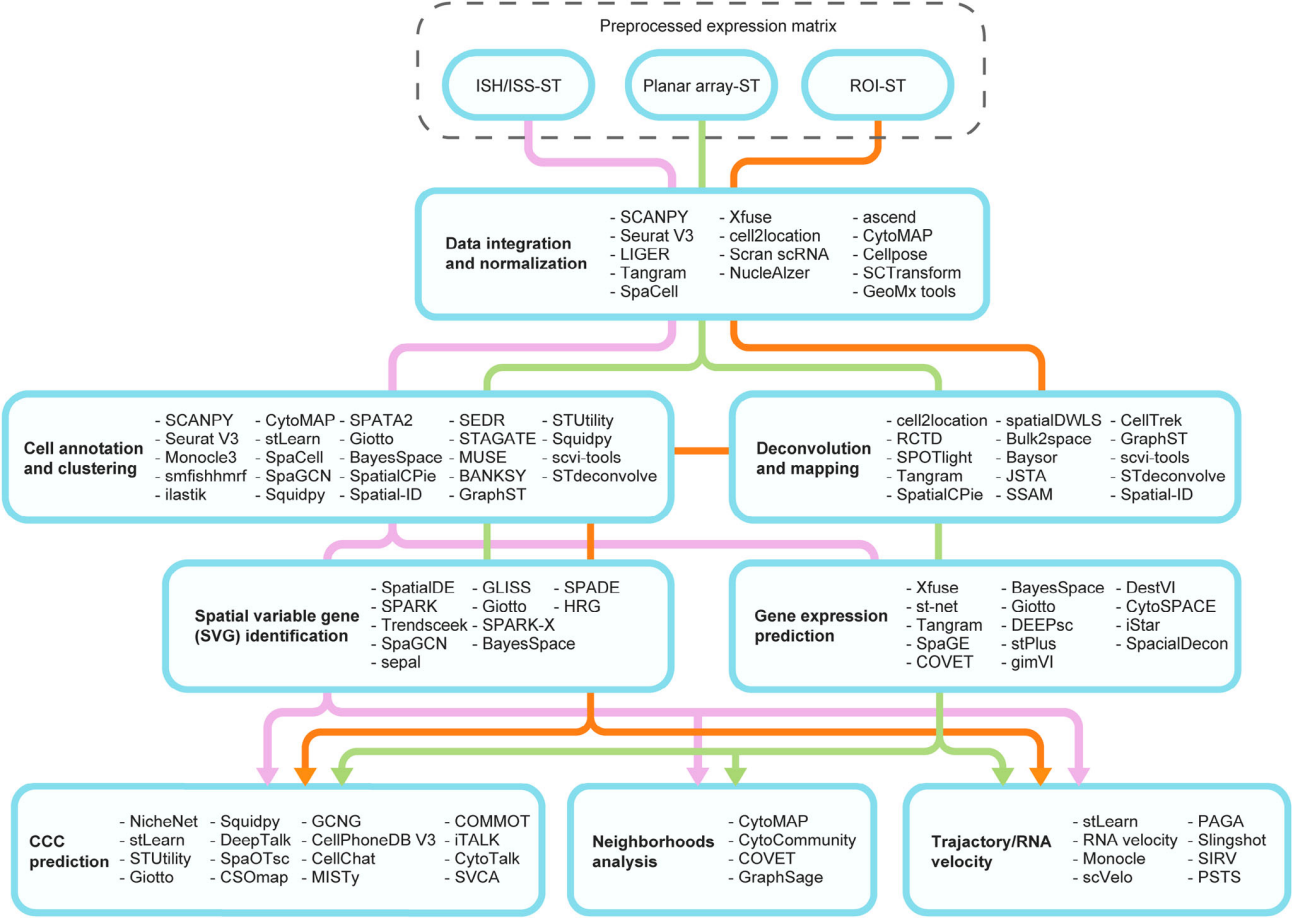
The spatial transcriptome data analysis toolbox. Graphical demonstration of typical ST data analysis pipeline based on three mainstream technical or strategical grounds (ISH/ISS‐ST, planar array‐ST, and ROI‐ST). Workflows start with preprocessed expression matrix and three different colored lines (light blue, pale green and orange) depict data analysis normally involved for three ST outputs respectively. The workflow directions are based on stepwise analysis approaches and each box represents a domain of spatial profiling methodology. *Notes*: The analysis toolbox is summarized based on literature searches and may not include all available methods. ISH, in‐site hybridization; ISS, in situ sequencing; ROI, region‐of‐interest.

Regardless of diversified data formats, gene detection efficiencies and spatial resolution derived from various ST platforms, in particular those from discrete ROI‐based ST technologies, the preprocessing for image registration and alignment, data binning needed for microarray‐ST as well as the cell segmentation used in ISH/ISS‐based ST, the mainstay of downstream analysis still shares a few in common and thus is summarized in a comprehensive but nonexhaustive list presented in Figure 2. The detailed methods and underlying algorithm principles can be referred elsewhere without detailed discussion in this section.^64^, ^65^, ^69^

Basically, microarray‐ST and ISH/ISS differ slightly from common scRNA‐seq data in that they contain extra spatial indices for individual data points generated across tissue‐covered regions being analyzed. Therefore, by taking advantages from computational methods developed for scRNA‐seq data analysis, many existing methods can be intuitively transferred and implemented such as Seurat V3 (integrated toolbox), SCTransform, Scran, and harmony for data integration/normalization^70^, ^71^, ^72^, ^73^, ^74^; Squidpy (Scanpy), monocle 3, and scvi‐tools for clustering and annotation^75^, ^76^; Trendsceek and HRG for SVG identification^77^, ^78^; CellPhoneDB and CellChat for cellular crosstalk profiling^79^, ^80^; and Slingshot, scVelo, and RNA velocity for transcriptional dynamics measures and cell state tracking (trajectory/RNA velocity).^81^, ^82^, ^83^ Simultaneously, inspired by efflux of available datasets within the community, by incorporating ST and histological information, researchers adopted various machine learning, topology‐based approaches and deep learning algorithms to continuously develop tools for ST analysis.^63^, ^64^, ^65^ Analysis frameworks including stLearn, Squidpy, Giotto, SPATA2, Tangram, STUtility, CytoMAP, Spacemake and others suitable for multitasking in ST data analysis.^84^, ^85^, ^86^, ^87^, ^88^, ^89^, ^90^, ^91^ Besides, many other bioinformatic tools are also established for fit‐for‐purpose analysis tools including LIGER, BayesSpace, SpatialDE, RCTD, cell2location, spatialDWLS, SpaGE, gimVI, SpaOTsc, SIRV, and many more summarized in Figure 2.^92^, ^93^, ^94^, ^95^, ^96^, ^97^, ^98^, ^99^, ^100^

### 4.3 Comparison of computational tools used across ST

Given the robust performance of data integration, normalization, and cluster identification using these ST‐adaptable tools,^101^, ^102^ many have been focusing on data enhancement, spatial clustering, spatial resolution enhancement, and cell type annotation/deconvolution as ample amount of ST data are realistically not down to single‐cell resolution (such like 55 µm in diameter in case of Visium polyA). A recent work systemically summarized 13 computational methods (conventional nonspatial methods: Louvain, Leiden and spatially designed: spaGCN, BayesSpace, stLearn, and many others) used for clustering of ST data.^103^ On leveraging their performance on clustering accuracy, spatial continuity, marker gene detection, scalability and robustness, the major determinant relies significantly on the spatial resolution of technologies per se. Taking clustering accuracy as the key parameter, at 55 µm (Visium), GraphST, SCAN‐IT, and BASS stand as the preferred methods; however, at single‐cell resolution (MERFISH), the best performing methods switch to CCST, SpaceFlow, and SCAN‐IT.^103^ Other side‐by‐side comparisons for spatial clustering have concluded that Seurat‐LVM, SpaGCN, and Seurat‐LV had overll the most accurate performance.^102^ Previously as the most extensive used STs were Visium polyA (spatial‐barcoded AT) and DSP (ROI‐based ST) both of which have limited spatial resolution for data interpretability to define cell types, many methods have been developed accordingly. Systematic comparisons were also conducted between methods giving the conclusion that some scRNA‐seq reference‐based methods (cell2location, CARD, RCTD, Tangram, and EnDecon) may outperform others.^101^, ^104^ Other approaches including also exist such like STdeconvolve, SMART and CARDfree that are scRNA‐seq reference‐free.^105^, ^106^, ^107^ With increasing resolution, including those with cellular or subcellular resolution (most ISH/ISS‐based), methods employing label transfer from scRNA‐seq, such as Spatial‐ID and JSTA are being applied.^108^, ^109^, ^110^ In addition, for imaging‐based ST reaching over single‐cell resolution, known genes are often predesigned into analytical panels and thus cell types are directly definable based on this.^27^, ^59^, ^111^ In real practice, it is also worth noting that for spatial‐barcoded ST with near single‐cell resolution, typical analytical points often require data binning to incorporate enough reads for profiling. However, the major challenge lies in that cells are irregular shaped disallowing precise transcripts allocation into designated cells. Under such scenario, spatial deconvolution remains as a standard tool for cell type inference but herein the concept is to take the cell type with the highest fraction for the cell bin being analyzed. Finding localized gene expression pattern (SVG) is another typical work implemented in spatial analysis wherein many have been developed and testified including regression‐based SpatialDE, Trendscreek, and SPARK‐X, multiple machine learning‐based spaGCN, sepal, GLISS, and many others.^63^, ^77^, ^95^, ^112^, ^113^, ^114^, ^115^ A recent benchmarking work was carried out evaluating eight SVG identification methods (SpatialDE, SPARK‐X, Giotto, MERINGUE, and others). Under multiparameterized comparison, they found acceptable proportion of genes were detected across SVG methods but with relatively low overlapping, however relative coexpression of those SVG across methods are generally matched.^116^ Under adjusted false discovery rate, SpatialDE, nnSVG, and MERINGUE are the top performers though certain issues still exist such as reproducibility (SpatialDE) and SVD prediction accuracy that exists almost across all methods except SPARK‐X and SOMDE.^116^ To boost the number of genes characterized via ST, another popular direction in ST method development is gene inference and by model construction using scRNA‐seq and high‐plex ST data, methods such as Tangram, spaGE, gimVI, iSpatial, and iStar are proven methodologies for this type of analysis.^89^, ^97^, ^101^, ^117^, ^118^ A recent work characterized 12 leading methods for gene expression imputation with ST datasets covering Visium polyA, Stereo‐seq, and Slide‐seq.^119^ Overall, methods incorporating spatial information generally have higher prediction accuracy and the newly developed graph neural network‐based method Impeller outpaced other competitors for gene imputation regardless of the ST platforms being used.^119^ Another added advantage of ST is to identify true interactors (cell–cell/gene–gene) under predefined spatial setting, a major advantage to apply in spatial biology since it gives histologically visible information that is completely lost in scRNA‐seq data. Those particularly involve tissue‐informed characterization of the signaling crosstalk (such as ligand‐receptor pairs) within direct or short‐ranged physical distance under cellular contexts. In theory, spatial cellular cross‐talk can be treated as interacting cell clusters often delineated via pathological annotation or within molecular‐informed cellular niches. Therefore, once a spatial count matrix is derived from particular histological regions (a subhistological cell clusters such as tumor epithelium‐enriched regions, tumor–immune interfaces, or tertiary lymphoid structures [TLS]), many existing approaches can be deployed. These mainly include conventional methods such as popular cell surface protein permutation‐based CellPhone DB and CellChat with proven performance in some benchmark studies.^79^, ^80^, ^111^, ^120^ intracellular gene–gene interaction‐based methods: NicheNet and CytoTalk^121^, ^122^ and more recently developed COMMOT using collective optimal transport.^123^ Besides, cell–cell communication analysis can also be conducted using probabilistic‐based and machine learning‐based tools: SVCA, GCNG, and MISTy.^124^, ^125^, ^126^ Similarly, most of spatial cellular fate dynamic tracking (trajectory‐based analysis) also employs well‐utilized scRNA‐seq tools such as stLearn, RNA velocity, scVelo, Monocle, Slingshot, PAGA,^81^, ^82^, ^83^, ^86^, ^127^ many of which are extensively cross‐compared^128^ and meanwhile others (SIRV) integrating ST with single‐cell also emerged.^100^ A note to take in is that for existing targeted spatial single‐cell technologies, trajectory analysis is often employed to explore specific cell or cell network alteration across various status (such as cross‐group comparison between normal and disease settings or drug responder versus nonresponder). Given the ST‐defined individual cells in local space (similar to those achieved via spatial phenotyping techniques such as high‐plex immunohistochemistry IHC and Imaging mass cytometry IMC), high‐ordered cellular architectures such as CNs, colocalization patterns, and local enrichment profiles can also be achieved.^66^, ^129^ Though systematically sparse and relying on individual computational efforts, some methods have been developed such as CytoMAP toolbox and recently developed CytoCommunity and GraphSage.^130^, ^131^ Moreover, we already see those applications being applied under a few research settings.^111^ Last, from our extensive experience, it is equally import to state that for most of ROI‐based ST such as widely used GeoMx DSP (pipelined in GeoMx tools),^84^, ^132^ though many analytical approaches can be potentially adopted such as ROI‐level clustering, SVGs, cellular decomposition, and trajectory analysis,^133^, ^134^, ^135^ the data exploration is rather context‐dependent and normally follows methods being used in bulk RNA‐seq analysis and explained in the above section. However, the in‐depth data covered by entire transcriptomics in ROI‐based ST such as DSP facilitate biological exploration using sophisticated tools such as WGCNA that relies on network topology to untangle gene regulatory modules associated phenotypes.^136^, ^137^

### 4.4 A conclusive remark toward bioinformatics tools in ST

From a user's perspective, our survey of spatial bioinformatics is yet not explicit and depending on the ST technologies or various computational tools, user experience‐based data benchmarking will still be in demand considering data formats as input in conjunction with thorough understanding of pathology and explainable biological phenomenon under investigation. We summarized the latest update of those bioinformatic tools that are available for open publics and their application in representative publications in Table 1. Though currently there are no generalizable standards as to what methods to be applied under a given condition, certain computational methods may emerge as the mainstream along with cumulating ST data publicly available. Powered by the ever‐increasing resolution down to single‐cell or even subcellular level, spatial deconvolution may be surpassed and under these resolutions, higher molecular capture efficiency may allow thousands of genes to be detected and quantified within a particular cell. Upon such, benchmarking on current and future computational methods may be reinstigated redefining our analytical paradigm for ST data.

**TABLE 1:** Representative bioinformatic tools frequently used for spatial transcriptomics.

Analysis	Method	Year developed	Main ST application	Application examples in ST
Data integration and normalization	Squidpy (Scanpy)	2022	Spatial‐barcoded ST, ISS/ISH‐ST	^138^
	Seurat V3	2019	Spatial‐barcoded ST, ISS/ISH‐ST	^71^
	LIGER	2023	Spatial‐barcoded ST	^139^
	Tangram	2021	Spatial‐barcoded ST, ISS/ISH‐ST	^89^
	cell2location	2022	Spatial‐barcoded ST	^93^
	SpaCell	2020	Spatial‐barcoded ST	^140^
	Xfuse	2021	Spatial‐barcoded ST	^141^
	stLearn	2020	Spatial‐barcoded ST	^142^
	Ascend			
	SCTransform	2019	Spatial‐barcoded ST, ISS/ISH‐ST	^143^
	Harmony	2019	Spatial‐barcoded ST,	^73^
	GeoMx tools		ROI‐ST	
Cell annotation and clustering	spaGCN	2020	Spatial‐barcoded ST, ISS/ISH‐ST	^114^
	BayesSpace	2021	Spatial‐barcoded ST, ISS/ISH‐ST	^94^
	stLearn	2020	Spatial‐barcoded ST, ISS/ISH‐ST	^142^
	BANKSY	2022	Spatial‐barcoded ST, ISS/ISH‐ST	^144^
	BASS	2022	Spatial‐barcoded ST, ISS/ISH‐ST	^145^
	SCAN‐IT	2021	Spatial‐barcoded ST, ISS/ISH‐ST	^146^
	STAGATE	2022	Spatial‐barcoded ST, ISS/ISH‐ST	^147^
	GraphST	2023	Spatial‐barcoded ST, ISS/ISH‐ST	^148^
	SEDR	2021	Spatial‐barcoded ST, ISS/ISH‐ST	^149^
Deconvolution and mapping	cell2location	2022	Spatial‐barcoded ST, ROI‐ST	^93^
	RCTD	2021	Spatial‐barcoded ST, ROI‐ST	^96^
	SPOTlight	2021	Spatial‐barcoded ST, ROI‐ST	^150^
	Tangram	2021	Spatial‐barcoded ST, ISS/ISH‐ST, ROI‐ST	^89^
	SpatialDWLS	2021	Spatial‐barcoded ST, ROI‐ST	^92^
	STdevonvolve	2022	Spatial‐barcoded ST, ROI‐ST	^106^
	CellTrek	2022	Spatial‐barcoded ST, ROI‐ST	^151^
	CARD	2022	Spatial‐barcoded ST, ROI‐ST	^105^
	JSTA	2021	ISS/ISH‐ST	^110^
	Bulk2space	2022	Spatial‐barcoded ST, ISS/ISH‐ST	^152^
Spatial variable gene (SVG)) identification	SpatialDE	2018	Spatial‐barcoded ST, ISS/ISH‐ST	^95^
	SPARK	2020	Spatial‐barcoded ST, ISS/ISH‐ST	^112^
	Trendsceek	2018	Spatial‐barcoded ST, ISS/ISH‐ST	^77^
	spaGCN	2021	Spatial‐barcoded ST, ISS/ISH‐ST	^114^
	sepal	2021	Spatial‐barcoded ST	^113^
	GLISS	2020	Spatial‐barcoded ST	^153^
	STAGATE	2022	Spatial‐barcoded ST, ISS/ISH‐ST	^147^
Spatial gene expression prediction	gimVI	2019	ISS/ISH‐ST	^154^
	iStar	2024		^118^
	Tangram	2021	Spatial‐barcoded ST, ISS/ISH‐ST	^89^
	SpaGE	2020	ISS/ISH‐ST	^97^
	COVET	2024	ISS/ISH‐ST	^155^
Cell–cell communication	CellPhoneDB v3	2020	Spatial‐barcoded ST, ISS/ISH‐ST, ROI‐ST	^79^
	COMMOT	2023	Spatial‐barcoded ST, ISS/ISH‐ST	^123^
	spaOTsc	2020	Spatial‐barcoded ST, ISS/ISH‐ST	^99^
	MISTy	2022	Spatial‐barcoded ST	^126^
	GCNG	2020	ISS/ISH‐ST	^125^
	stLearn	2020	Spatial‐barcoded ST	^142^
	DeepTalk	2024	Spatial‐barcoded ST, ISS/ISH‐ST	^156^
	CellChat	2021	Spatial‐barcoded ST, ISS/ISH‐ST, ROI‐ST	^80^
	SpaTalk	2022	Spatial‐barcoded ST	^157^
Trajectory and RNA velocity	stLearn	2020	Spatial‐barcoded ST	^142^
	Monocle	2017	Spatial‐barcoded ST	^158^
	PSTS	2023	Spatial‐barcoded ST	^142^
	SIRV	2021	ISS/ISH‐ST	^159^
	PAGA	2019	Spatial‐barcoded ST, ISS/ISH‐ST	^127^
	scVelo	2020	Spatial‐barcoded ST, ISS/ISH‐ST	^81^
Neighborhoods analysis	CytoMAP	2020	Histo‐cytometry	^85^
	CytoCommunity	2024	ISS/ISH‐ST	^130^
	COVET	2024	ISS/ISH‐ST	^155^

## 5 ST‐DRIVEN RESEARCH IN TRANSLATIONAL ONCOLOGY

### 5.1 A foresight on ST in cancer research

**FIGURE 3:**
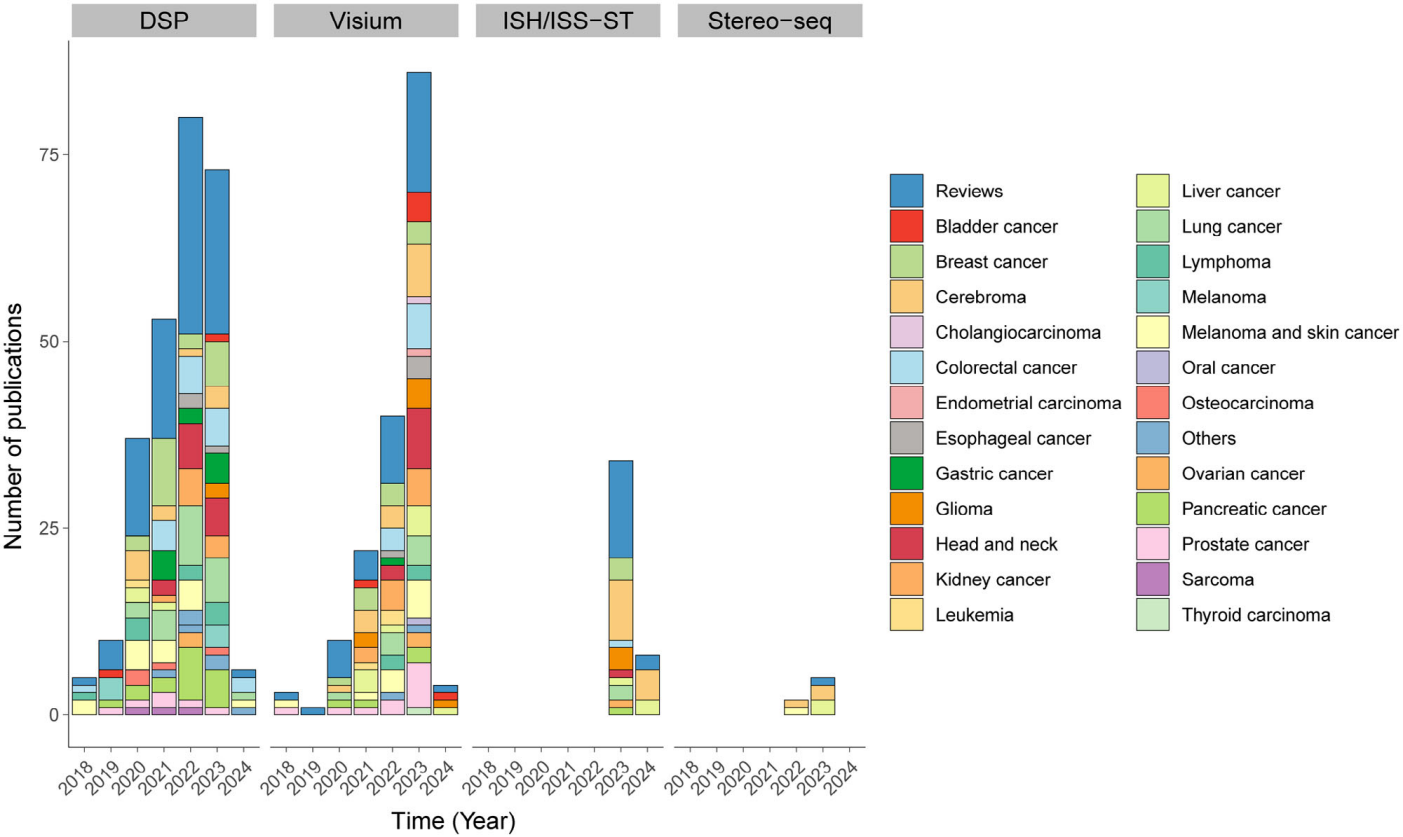
Overview of growth pattern of spatial transcriptome application in cancer research. Data are summarized in stacked bar charts and collected between 2018 and 2024 (current) with mainstream platforms commercially available (DSP, Visium, MERSCOPE, CosMx, Xenium, Stereo‐seq). Major cancer types are color‐annotated and reviews are separately categorized. ISS/ISH‐based ST (MERSCOPE, CosMx, and Xenium) are compiled together in one group and others are listed separately. *Notes*: Analysis is based on manual searches and may not include all related publications. Development of bioinformatic tools and abstracts are purposely excluded.

The way to study tissue oncogenesis has already tweaked the research paradigm, in which much is influenced by ST. This is simply because tumors on the whole reside in a changing ecosystem often referred as tumor microenvironment (TME), a complex milieu that opens to questions such as inter/intratumor heterogeneity (ITH), spatial cellular context‐dependent mechanisms and predictive, prognostic, or therapeutic biomarker identification based on high‐plex spatial molecular information. Some have already been deciphered using dissociation‐based techniques (single‐cell multiomics) bearing potentials to translate into clinics,^160^ but many more are yet to be unveiled. We foresee promises being accelerated by integrating high‐plex spatial omics with conventionally pathological techniques and thereby overview the current progress in this field. Our discussion is much toward the available technologies utilized throughout research and commercial institutions and summarized under such basis (Figure 3). Those mainly include but not restricted to Visium‐ST, Stereo‐seq, GeoMx DSP, and ISH/ISS‐based techniques (MERSCOPE, Xenium in situ, and SMI) and largely a surge is witnessed in the past few years.^49^, ^161^, ^162^ Another point to add is that we purposely focus on research adopting ST as the major exploratory tool throughout their studies, highlighting the added benefit from this technical point of view. Since low‐resolution ST are already widely used, we presented oncology‐related application using ST with near/true single‐cell resolution and summarized in Table 2.

**TABLE 2:** Application of near single‐cell spatial transcriptomics in oncology.

ST technology	Cancer type	Year	Multiplexity	References
MERFISH	BC	2023	500	^163^
Stereo‐seq	CRC	2024	Transcriptome‐wide	^164^
SMI, Xenium MERFISH	OV	2023	SMI: 960 Xenium: 280 MERFISH:140	^165^
MERFISH	HCC	2023	50	^166^
MERFISH	LC	2024	479	^167^
MERFISH	GBM	2021	135	^168^
Stereo‐seq SMI	HCC	2024	Transcriptome‐wide SMI: 960	^169^
Stereo‐seq	LC	2023	Transcriptome‐wide	^170^
Stereo‐seq	CRC	2023	Transcriptome‐wide	^171^
Stereo‐seq	HCC/ICC	2023	Transcriptome‐wide	^172^
Stereo‐seq	CSCC	2022	Transcriptome‐wide	^173^
Stereo‐seq Molecular Cartography	Melanoma	2022	Stereo‐seq: Transcriptome‐wide Molecular Cartography:	^174^
Stereo‐seq	CRC	2023	Transcriptome‐wide	^175^
SMI	OV	2024	960	^176^
SMI Xenium	HGG	2023	SMI: 960 Xenium: 339	^177^
SMI	HNC	2023	SMI: 960	^178^
Xenium	GBM	2024	358	^179^
Xenium	LC	2023	302	^29^
Xenium	BC	2024	280	^111^
Xenium Visium HD	CRC	2024	422 Transcriptome‐wide	^59^
Xenium	BC	2023	280	^180^
Xenium	GBM	2023	298	^181^
Slide‐seq V2	NBM	2024	Transcriptome‐wide	^182^
Slide‐seq V2	PC	2023	Transcriptome‐wide	^183^

*Notes*: References only incorporate real experiments using ST technologies excluding bioinformatics tool development, ST technology validation, reviews, or publications using public ST data.

Abbreviation: BC, breast cancer; CRC, colorectal cancer; OV, ovarian cancer; HCC, hepatocellular carcinoma; GBM, glioblastoma; LC, lung cancer; ICC, intrahepatic cholangiocarcinoma; CSCC, cervical squamous cell carcinoma; HGG, high grade glioma; HNC, head and neck cancer; NBM, neuroblastoma; PC, prostate cancer.

#### 5.1.1 Mechanistic elucidation in heterogenous cancer tissues

One favorable advantage of ST is its compatibility for both archived FFPE and FF tissues and in many circumstances only small handful of tissue is required as analytical inputs granting flexible fit‐for‐purpose experimental designs. In breast cancer, an active application field of ST, Andersson et al.^184^ used ST to find infiltration niche of CXCL10+ M2‐like macrophages with IFIT1+ T cells with HER2‐positive breast tumors yielding tertiary lymphoid‐like structures as a universal indicator across tissue types. Using ST combined with single‐cell T cell receptor‐seq, Mao et al.^185^ elucidated spatial‐driven M1/M2 characteristics associated with breast tumor that orchestrate with cancer‐associated fibroblasts (CAFs) to form immunosuppressive microenvironment. The spatially informed breast cancer transcriptome obtained via ST also reveals an imbalance in oxygen distribution within claudin‐low tumor, with hypoxia in the tumor center and normoxia in the periphery.^186^ Another advantage of ST is to investigate pathogen‐host responses where regions of infection can be precisely spotted.^187^ Galeano Nino et al.^188^ combined multiple spatial techniques with scRNA‐seq to map the spatial, cellular, and molecular interactions of host cells within the TME, revealing the presence and dynamics of intratumoral microbial communities and their potential impact on tumor heterogeneity. Similar works were also carried out using ROI‐ST (GeoMx‐DSP) to uncover bacteria burdens associated with lung cancer epithelium wherein Wnt/β‐catenin, HIF1A and VEGFA‐related signaling were mechanistically identified.^189^ In immunotherapy‐favored cancers, using ROI‐ST, a study involving 152 non‐small cell lung cancer (NSCLC) patients revealed that the spatially enriched 163+ tumor‐associated macrophages (TAM) in the TME are associated with immunotherapy resistance, driven by the upregulation of CD27, ITGAM, and CCL5 expression within the tumor cavity.^190^ Using joint scRNA‐seq and ST to delineate oncogenic transcriptional programs, Zhu et al.^191^ found that UBE2C+ cancer cells during the lung adenocarcinoma (LUAD) invasion, as a progression hallmark companied by multiple localized immune cell alteration and activated TGF‐β signaling. By dissecting TME into tumor and immune‐enriched areas, Zhang et al.^133^ established a ST landscape of NSCLC with brain metastasis, wherein reduced antigen presentation, B/T cell function and reprogrammed neutrophils and M2‐like macrophages, immature microglia, and reactive astrocytes were observed. Recently, Wang et al.^192^ elucidated spatial transcriptome scale molecular features during LUAD tumor progression and the establishment on holistic tissue architecture captured major events in hypoxia‐induced macrophages and other molecular characteristics in certain subtypes. In melanoma, comprehensive spatial exploration established mechanistic hallmarks within tumor precursor regions and tumor‐stomal boundary that involve gradient cytokines to stimulate immune cell recruitment.^193^ In tumors bearing less mutational burdens with complex TME with immune checkpoint inhibitors (ICIs) often being ineffective, Liu et al.^194^ combined ST with scRNA‐seq and multiplex immunofluorescence staining, revealing the existence of a tumor–immune barrier structure in hepatocellular carcinoma (HCC): a spatial niche composed of SPP1+ macrophages and CAFs near the tumor border. Mechanistic deciphering of this spatial niche revealed that a hypoxic microenvironment promotes SPP1+ macrophages as an advert regulator for effective check point blockade.^194^ More recently, Sun et al.^195^ established a comprehensive HCC primary and metastatic landscape wherein they used ROI‐ST to analyze Wnt‐mutation (Wnt‐mut) associated spatial transcriptome and found upregulated exhausted T cells associated with mutant phenotypes and wide‐type Wnt (Wnt‐wt) had enriched iCAF population. The same group also applied subcellular resolved ST (Stereo‐seq) to identify key regulatory events at the tumor–immune interface that involve trafficking of amloid A1 and A2 and activation of tumor‐stemming JAK–STAT3 via CXCL6.^172^ Zooming into the tumor and invasive margins, they found increasing patterns of angiogenetic signal, extracellular matrix remodeling and TP53 activity in Wnt‐wt tumors.^195^ In pancreatic ductal adenocarcinoma (PDAC), single‐cell transcriptomics combined with ROI‐ST covering the whole transcriptome delineated neoadjuvant chemotherapy and radiotherapy (RT)‐associated spatiotemporal dynamics of treatment‐refractory patients, a mechanism mediated by three multicellular communities, that are each reprogrammed.^196^ Another study incorporated ST to investigate treatment‐associated spatiotemporal changes in different therapeutic arms under neoadjuvant settings.^197^ They discovered tumor‐intrinsic and transitional cellular programs mediated via TIGHT+ exhausted and regulatory T cells and NECTIN‐regulatory in tumor‐proximal niches highlighting TIGHT–NECTIN axis as potential targets.^197^ A recent study incorporated multiparameterized spatial single‐cell proteomics and ROI‐ST to elucidate mechanistic linkage in PDAC patients stratified by homologues recombinant deficiency (HRD).^198^ They discovered tumor‐infiltrating macrophages that are elevated in response to oncogenic transformation and HRD status highlighting a potential therapeutic target CD52 in treating PDAC patients in combination with PARP inhibitors (PARPi).^198^ In gastrointestinal cancer, using ROI‐ST on FFPE samples, an early work demonstrated the utility of the technology to profile biomarkers in anti‐PD‐1 antibody driven TME alteration in colorectal cancer (CRC).^199^ Qi et al.^200^ used scRNA‐seq and ST to identify and orthogonally validate a tumor‐associated FAP1+ fibroblasts and SPP1+ macrophages in CRC, a mechanism involving chemerin, TGF‐β and IL‐1 to form immune‐resistant cellular niches with impaired T cell functioning. In gastric cancer (GC), Kumar et al.^201^ used ROI‐ST to cross‐validate INHBA+ CAF and tumor‐expressing KLF2 in the diffused subtype complimenting their finding from scRNA‐seq. In gynecological cancer, Yeh et al.^202^ recently applied ISH‐ST to uncover spatial alterations in high‐grade serous ovarian cancer (HGSC), wherein distinct infiltrated T/nature killer cellular states were spotted in subset of treatment sensitive tumor epithelium collaborating with transcriptional change in PTPN1 and ACTR8. In another application scenario, Liu et al.^203^ tracked spatiotemporal dynamics of esophageal squamous cell carcinoma from precancerous lesions to low‐grade/high‐grade intraepithelial neoplasia using ROI‐ST on transcriptome‐wide scale capturing major events at tumor sites mediated through inversely correlated TAGLN2 and CRNN as progressive hallmarks. In urological cancers, a recent work incorporating ST and scRNA‐seq identified PDGFRα+/ITGA11+ fibroblasts that mediate lymphovascular invasion and lymphatic metastasis via ITGA11–SELE ligand‐receptor crosstalk in early stage bladder cancer.^204^ In brain cancers, Vo et al.^205^ used Sonic hedgehog medulloblastoma derived organoid (SHH‐DPOX) to resolve cellular heterogeneity within the TME in response to Palbociclib treatment (a CDK4/6 inhibitor). Using Visium ST they spotted species lineage associated coexpression at the immune‐infiltration regions and Palbociclib‐treated models induced regression of clonogenicity of MB tumor together with activated neurodifferentiation in the tumor center but not at the tumor–immune boundary.^205^ More importantly, in the drug‐treated group, a spatially niche was also identified coordinated expression of astrocytes and tumor‐associated microglia together with tumor‐infiltrating macrophages (TMA) suggesting a functional interplay between them.^205^ Likewise, many other works used ROI‐ST to target specific regions within different cancer tissues (TLSs or perirenal fat enriched regions) to elucidate transcriptional mechanisms linking with disease prognosis.^134^, ^206^ Many more can be referred in recent reviews.^4^, ^161^, ^207^

Toward this end, however it is still well‐worth to mention that with ST technologies being constantly developed and improved, the real‐world application scenarios are much beyond early prototyping studies where model systems were often used for proof‐of‐concept studies and from extensive experience including ours. On top of this, due to the heavy investment upfront to generate large scale data across patient cohorts and intensive computational efforts for data integration (especially single‐cell spatial data that easily generate tens of millions of cells), more systemic works will still be needed. This is particularly indispensable when researching under such heterogenous tumor molecular mechanistic contexts. Those include some preliminary works being carried out by us and others using cutting‐edge technologies such as Xenium in situ, CosMx, and MERSCOPE as in their commercial forms and with propagate in short time.^27^, ^28^, ^166^, ^169^

#### 5.1.2 Empowering biomarker profiling using advanced ST in translational oncology

Of more clinical relevance, under many experimental contexts, the ultimate goal following mechanistic elucidation biomarker discovery that enables ultimate clinical implementation. This is significantly inspired by evolving tumor biology and the rapid progress in cancer drug development and their associated resistance mechanisms not only through tumor‐centric targeting approaches (modulation of cancer cell plasticity) but also versatile strategies involving microenvironment modulation.^208^, ^209^ Nowadays, the widespread application of machine learning/deep‐learning assisted by digital pathology in fusion with ST has emerged as a novel tool toward this direction. The multidimensional layer of omics embedded in situ brings unforeseen analytical potentials to catalyze novel biomarker translation. Leveraging ST profiling across whole‐slide histopathological images, deep learning algorithms can further develop meticulously trained models capable of capturing tumor biomarkers of clinical significance.^210^, ^211^ As said before, at this stage, most of ST‐based technologies are yet expensive to scale up in sample number to allow systemic profiling, but some especially those supporting FFPE or tissue microarray (TMA) application are in play since for those, clinical metadata are often at hand.

On this ground, we and others have already started to attempt in this field. TLSs, an indicator within the TME often associates with favored prognosis but in‐depth studies are lacking. Gan et al.^212^ applied ST to unveil TLS associated expression signatures in combined hepatocellular–cholangiocarcinoma (cHCC–CCA) patients and generated a scoring system considering spatial distribution of TLSs where intratumor TLSs (iTLS) stands as a predictive indicator for prognosis. Under another setting, using ROI‐ST, Kiuru et al.^213^ profiled melanoma TME and identified S100A8 expressed on keratinocytes as an early oncogenic biomarker and validated the finding in larger cohorts. Another systemic work using Visium ST profiled the heterogenous stroma TME in a set of HGSC.^214^ Their findings, arising from the complex TME identified a subset of CAF at the tumor‐stroma interface with significant intercellular crosstalk of APOE–LPR5 as a predictive biomarker for short‐term survival.^214^ Similarly, a recent work focusing on cHCC–CCA also employed Visium ST to discover TLS‐associated gene expression pattern that predict disease prognosis.^212^ Based on the ST‐derived data, they developed a TLS score by leveraging the contribution of either intratumor or extratumor regions (iTLS and eTLS) wherein the iTLS had better prognostic values that were not observed in eTLS counting, again stressing the needs of taking spatial parameters for prediction model generation.^212^ Monkman et al.^215^ applied ROI‐ST to discover T cells and macrophage‐dominated immune traffics that potentially relate to ICI responses in NSCLC. They built spatially chartered proteogenomic biomarker panels using sPLS‐DA model to discriminate ICI response and overall survival.^215^ These phenomena are also reflected on some preliminary spatial biomarker profiling works being carried out in ICI‐related studies in other thoracic and GCs.^216^, ^217^ Notably, works from above studies acquired extensive resources from archived FFPE samples in which many were conducted in a TMA format suggesting a trend in practice of those ST technologies. Likewise, in our hands, using ROI‐ST, Guo et al.^136^ explored 45 mismatch repair‐deficient endometrial cancer (MMRd‐EC) finding a 14‐gene biomarker signature associated with tumor cells that defines three EC subtypes with varying CD8 T cells infiltration status. In the more deadly lung cancer subtype (small cell lung cancer, SCLC), ST was applied to define TME‐based molecular subtypes. In a retrospective study, Yang et al.^218^ used ROI‐ST to discover two immune microenvironment‐defined molecular subtypes (ID: immune‐deficient and IE: immune‐enriched) in primary SCLC (TMA cohort of 29 patients) using transcriptome‐wide spatial profiling and found the clinical utility of this TME‐based subtyping in predicting patient survival outcomes and response to immunotherapy. Using the same ST technique, they used multiple ROI selection strategy to evaluate ITH of 25 SCLC patients using DEPTH algorism and identified three transcriptional subtypes featured by distinct molecular mechanisms.^219^ More importantly, this classification correlated with CD8 T cell infiltration status and by grouping patients based on spatially defined ITH scores, they found high‐plex (HC) and low complex (LC) defined patients that differentiate between clinical outcomes.^219^

Interestingly, we witnessed a gradual growing tendency of spatial multiomics being incorporated into clinical trial‐based studies undertaken by research communities and ourselves (data not to disclose), though most are retrospectively designed. For the sake of functional interpretation, these are mainly informed via spatial proteomics tools.^131^, ^220^ However, exploratory works in novel drug development (netrin‐1),^160^, ^221^ investigator initialed trials, as well as real‐world studies incorporating ST are gradually emerging and some studies have already become low‐hanging fruit. The forward‐looking assumption is made since the true spatial single‐cell ST performed using clinically archived samples is tangible. This may be gradually incorporated into careful designed clinical project pipelines wherein 10−100 proteins can be characterized under single‐cell spatial resolution, but beyond that, have extended analytical scope since the transcriptional state of over 10,000 genes at spatial single‐cell resolution is now in reach. The preliminary works on breast cancer have already shown promises as the growing prosperity of neoadjuvant therapies in breast cancer allows trial designs and biopsy sampling longitudinally. For example, in a phase I/II single‐arm study assessing pembrolizumab + sequential RT under neoadjuvant settings in triple‐negative breast cancer (TNBC), using multiplex IHC (mIHC), researchers examined 40 proteins to define cellular lineages within the TME and mapped all high‐ordered cellular niches (defined as districts) in space finding key regulatory mechanisms predisposed or induced in response to therapies.^131^ In a multicentric randomized study (NeoTRIP clinical trial, NCT02620280), researchers explored the effect of atezolizumab + chemotherapy versus chemotherapy alone in early TNBC using a longitudinal approach.^220^ They identified major events triggered via TCF1+ CD8 T cells and MHCII+ cancer cells as drug sensitive predictors and other immune‐dictated coregulatory multicellular modules involving B cells and granzyme B+ cytotoxic T cells underscoring their values in personalized clinical decision making.^220^ These recent works, although being exploratory, may be a weathercock in the coming future under clinical settings. Nevertheless, it is equally important to stress that, the success path for cancer biomarker identification is wrapped in a coordinated delivery package querying into omics‐based profiling from upstream and corroborating in independent cohorts and relevant functional assays downstream.

### 5.2 Streamlining translational oncology via ST from bench to clinics

The plethora of new ST constantly drives the growth of translational oncology gradually redefining our understanding into this field. However, we still see the progress made in current era as being a puberty that is yet to flourish in the future. Similar to the development curve witnessed in dissociation‐based single‐cell omics whereby application is moving into clinical settings evidenced by some novel‐conceptualized clinical trials. For example, EXALT‐1/2, which are hematological malignancies‐oriented interventional study, incorporated scRNA‐seq and AI‐driven drug screening to guide clinical decision‐making.^160^, ^222^ A single‐arm proof‐of‐concept phase II trial combining tumor‐targeting B‐RAF inhibitor and MEK1/2 inhibitor with PD‐1 inhibitor (PDR001) also launched in metastatic CRC utilizing scRNA‐seq to analyze pretreatment and on‐treatment biopsy samples.^160^ These and a few more prospective studies point at a promising potential of integrating novel omics into clinical settings. One key advantage of spatial multiomics is their tight coherence with standard pathology, a major benefit for data exploration and interpretation perspective under controlled settings. This is somewhat impractical using conventional dissociation based single‐cell techniques since most are relying upon fresh tissues, which are difficult to manipulate. More importantly, since spatial organization of cells are more important to decipher biological questions, the spatially coordinated cells bear eminent potentials for translational medicine, similar to what was observed in ICI modulation wherein spatial PD‐L1 distribution and abundance are key determinants for therapeutic responses in multiple cancers.^223^ Other successful biomarkers such as “immunoscore” derived from quantitative measurement of tumor‐infiltrated CD3+/CD8+ T cells has already placed into the application frontline in clinics in CRC and potentially many others.^224^ In addition, the development of novel checkpoints or drug combinations require investigation of TME in a depth of field to underpin deterministic biological mechanisms.^225^ These are already evidenced at phenotypical level. Such as oncolytic virus (OV) being tested in clinics. Linking OV with immune‐activation in recurrent glioblastoma has been observed with novel CAN‐3110, an oncolytic herpes virus (oHSV).^226^ Other supporting evidence also revealed altered neutrophil‐to‐lymphocyte ratio during OV (H101 an oncolytic adenovirus) pretreatment as good prognostic indicator in advanced refractory HCC.^227^ Moreover, the recent success of antibody–drug conjugates (ADC) in many late‐stage solid tumors have also ushered the needs of patient stratification biomarkers^228^ A few good examples include the success application of T‐DXd (a HER2–ADC) that is being approved for metastatic HER2+ breast cancer and sacituzumab govitecan (SG), a trophoblast cell‐surface antigen 2 (TROP2)‐targeting ADC.^228^ Interestingly, ample amount of clinical evidence suggest that the bystander effect induced by those ADC often reprograms TME and such include T‐DXd being effective in HER2‐low patients and SG nondiscriminatively targets tumors with low‐TROP2 expression.^228^ Since many of these ADCs are being explored with combinatorial regimes with other drugs such as Atezolizumab (anti‐PD‐L1 antibody) in metastatic BC (mBC, NCT04740918) and late‐stage/locally advanced urothelial cancer (la/mUC) with combination of enfortumab vedotin (a Nectin‐4 AD**C)** with pembrolizumab (anti‐PD‐1 antibody), both of which demonstrated promising clinical benefits, adding high‐plex spatial data would be beneficial to address TME‐related mechanisms.^229^ This would ultimately deliver extra clinical benefit via balancing immunogenicity by removing undesirable adverse effects while inflaming or reverting immune excluded tumor stands as the main battlefield in oncoimmunology.^230^ Beyond those facts, the multiparametrized data obtained with spatial dimension certainly have added values for patient stratification and may direct novel biomarker identification in a single or multimarker collaborated manner.^231^

**FIGURE 4:**
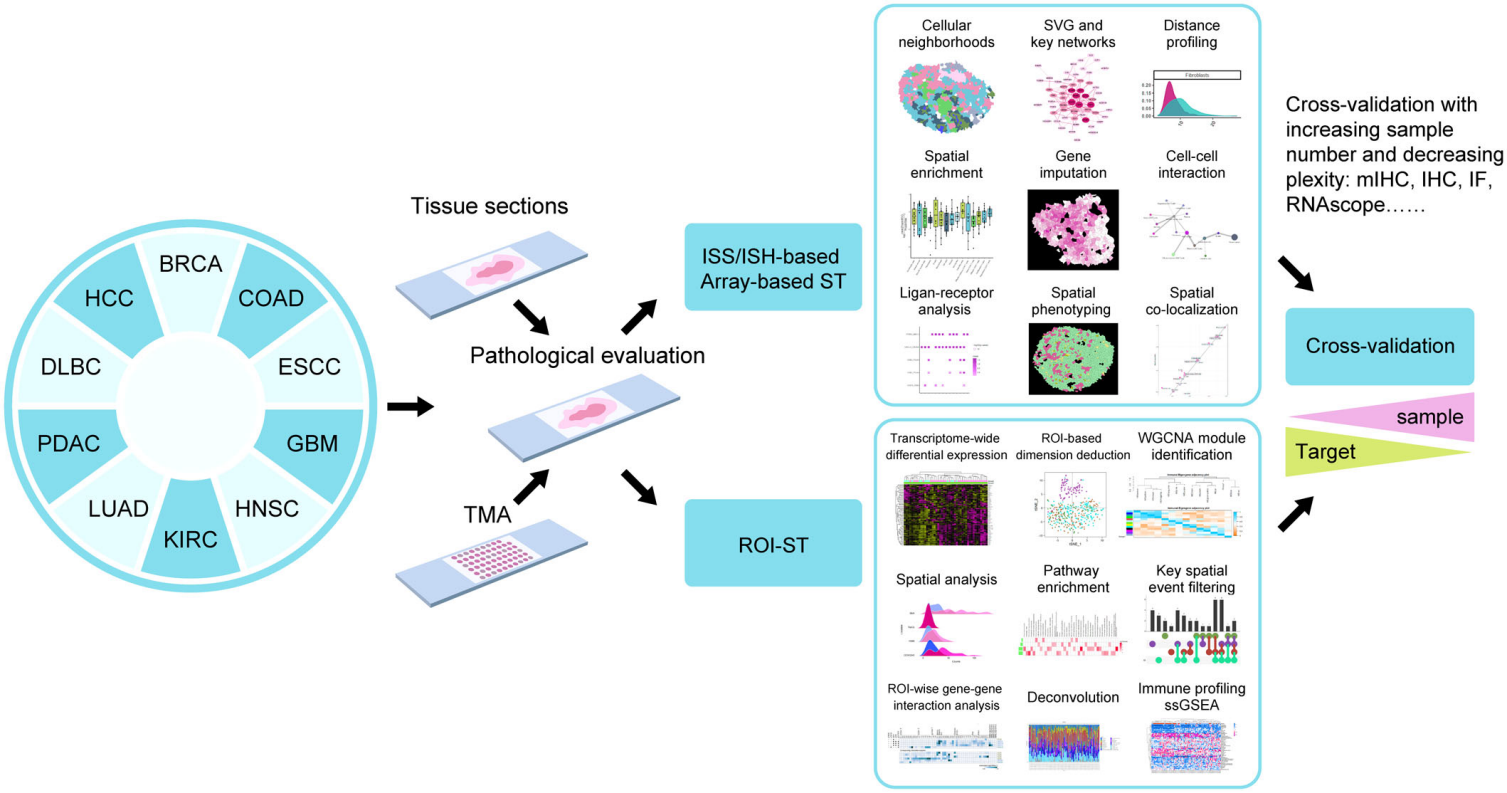
Proposed biomarker development pipeline using spatial transcriptome. Schematic illustration of biomarker profiling workflow based on ST. Cancer types are listed on the left for demonstrative purposes (see abbreviations). Sampling strategies are divided into full tissue sections and tissue microarrays (TMA) depending on ST technologies as exploratory tools (ISS/ISH‐ST, array‐based ST, and ROI‐ST). bioinformatic explorations (listed in boxes) are grouped accordingly to different ST technologies being applied. Downstream validation is for demonstration but involves increasing number of samples and decreasing number of targets. BRCA, breast carcinoma; COAD, colon adenocarcinoma; ESSC, esophageal carcinoma; GBM, glioblastoma multiforme; HNSC, head and neck squamous cell carcinoma; KIRC, kidney renal cancer; LUAD, lung adenocarcinoma; PDAC, pancreatic ductal carcinoma; DLBC, lymphoid diffuse large B‐cell lymphoma; HCC, hepatocellular carcinoma.

The developed ST techniques in current era are already reaching into the single‐cell level with ultrahigh‐plexity conducted in many prototype studies allowing true single‐cell spatial phenotype identification with simultaneously thousands of transcriptomic information aligned. While determining pivotal cell subtypes such as tumor‐reactive proimmune cells, rare malignant cell clones with pluripotency and plasticity and other key regulatory cells within the TME is becoming possible, careful design of experiments or orthogonal validation across ST platforms for fit‐for‐purpose analysis lay ahead of the biological questions. Herein, we propose a systemic workflow based on our user's experience to research communities (Figure 4). Practically, a balance between the cost efficiency and layers of data dimension (spatial resolution and target numbers) to be acquired need to be compromised. It may not be a huge burden to query into mechanism‐related issues since not many samples are required, but more for translational purposes. In addition, considering the timeline for research output, possibility of sample procurement, preexperimental QC measures, using clinical archived samples may be a wider future direction and many ST are now compatible with this type of samples. Therefore, a schema can start with a rather small sample set within which each assigned phenotypic group contains 3−6 samples for single‐cell ST technologies (planar array‐ST and ISS/ISH‐based) and these samples sometimes can be pooled into one glass‐slide to save costs or conducted using TMA in some circumstances. This pan‐tissue exploratory method allows limit‐free analysis for cell–cell association, biomarker‐specific cell type identification and orchestrated cell community identification based on manually defined cell types within contexts. Selected candidates can then be cross‐compared against RNAscope, mIHC, cyclic IF, and IMC for high‐plex profiling and resultant conclusions can be extrapolated and cross‐validated using clinically benchmarking technologies such as IHC and FISH. Meanwhile, deeper mechanistic analysis can be done via ROI‐ST targeting cells at specific ROI. The alternative strategy to deploy involves thorough evaluation of on‐study samples by experienced pathologists and for biomarker profiling, patients in each assigned metadata group often start with at least 10 samples and are made into TMA formats. Under such a background, using ROI‐ST, particular ROIs can be analyzed across patients within a well‐controlled environment under pathological assistance (tumor‐centric, tumor–immune interface or a particular cell type with TME). This often supports transcriptome‐wide discovery and parallel mechanistic elucidation and one can use well‐defined deconvolution methods to deduce major cell type alteration in abundance. Orthogonal validation using RNAscope, mIHC, cyclic IF, and IMC will subsequently be required in follow‐up analysis and cross‐validation using IHC or FISH on expanded cohorts can follow. However, in both circumstances, full pathological engagement is paramount throughout a study to lead to successful interpretation.

## 6 CONCLUSIVE REMARKS AND FUTURE DIRECTION

Finally, our conclusive remarks are drawn in view of the current progress made on technical development as well as translational oncology being conducted. We anticipate a further improvement of existing technologies with joint multidimensional omics in real practice and these include high‐plex spatial proteogenomics, epigenetics assayed via transposase‐accessible chromatin, spatial T/B cell receptors (Spatial V(D)J recombination), and metabolomics obtained at bona‐fide single‐cell or even subcellular resolution.^232^ Alongside with these layers of spatial information, reconstruction of tissue architecture in three‐dimensional using ST is also underway.^233^ Moreover, the growing needs of researching into intratumoral microbiota open a new avenue to explore since many microorganisms are found in solid tumors that play either cancer‐initiating or inhibitory roles.^234^ However, their exact mechanisms cannot be elucidated without application of ST and tracking the colocalization pattern of diversely distributed microbes within host tissues holds promises to address those questions. Some of those technologies are being developed such as spatial‐host‐microbiome sequencing (SHM‐seq) or many other ISS‐based methods.^235^, ^236^ More importantly, alongside with broader affordability and data accessibility, benchmarking on technologies or bioinformatics are always needed as references for preexperimental consideration. Aggregating the multilayered spatial omics can ultimately become the analytical keystone undoubtfully in cancer research, a gap to be filled just around the corner.

## AUTHOR CONTRIBUTIONS

*Conceptualization, data collection, and writing original draft*: Nan Wang. *Data analysis, data collection, and writing original draft*: Weifeng Hong. *Presentation, data collection and writing original draft*: Yixing Wu. *Guidance and review—editing*: Zhe‐Sheng Chen. *Supervision and review*: Minghua Bai. *Conceptualization, review—editing and supervision*: Weixin Wang. *Supervision, review—editing and fundings*: Ji Zhu. All authors have read and approved the final manuscript.

## CONFLICT OF INTEREST STATEMENT

N. W. and W. X. W. are full‐time employees of Cosmos Wisdom Biotech Co. Ltd. The commercial participant offers spatial multiomics as standard services. Author Zhe‐Sheng Chen is an Editorial board member of Medcomm. Author Zhe‐Sheng Chen was not involved in the journal's review of or decisions related to this manuscript. All authors declare no competing interests.

## ETHICS STATEMENT

There is no ethical approval related to this review and no human and animal data are included.

## Data Availability

All data collections including all technical aspects and research references are manually curated, presented in relevant figures and available upon request.

## References

[1] Maman S , Witz IP . A history of exploring cancer in context. Nat Rev Cancer. 2018;18(6):359‐376. 29700396 10.1038/s41568-018-0006-7

[2] Bressan D , Battistoni G , Hannon GJ . The dawn of spatial omics. Science. 2023;381(6657):eabq4964. 37535749 10.1126/science.abq4964 PMC7614974

[3] Robles‐Remacho A , Sanchez‐Martin RM , Diaz‐Mochon JJ . Spatial transcriptomics: emerging technologies in tissue gene expression profiling. Anal Chem. 2023;95(42):15450‐15460. 37814884 10.1021/acs.analchem.3c02029 PMC10603609

[4] Elhanani O , Ben‐Uri R , Keren L . Spatial profiling technologies illuminate the tumor microenvironment. Cancer Cell. 2023;41(3):404‐420. 36800999 10.1016/j.ccell.2023.01.010

[5] Yue L , Liu F , Hu J , et al. A guidebook of spatial transcriptomic technologies, data resources and analysis approaches. Comput Struct Biotechnol J. 2023;21:940‐955. 38213887 10.1016/j.csbj.2023.01.016 PMC10781722

[6] Asp M , Bergenstrahle J , Lundeberg J . Spatially resolved transcriptomes‐next generation tools for tissue exploration. Bioessays. 2020;42(10):e1900221. 32363691 10.1002/bies.201900221

[7] Wang N , Li X , Wang R , Ding Z . Spatial transcriptomics and proteomics technologies for deconvoluting the tumor microenvironment. Biotechnol J. 2021;16(9):e2100041. 34125481 10.1002/biot.202100041

[8] Park HE , Jo SH , Lee RH , et al. Spatial transcriptomics: technical aspects of recent developments and their applications in neuroscience and cancer research. Adv Sci (Weinh). 2023;10(16):e2206939. 37026425 10.1002/advs.202206939 PMC10238226

[9] Zhou R , Yang G , Zhang Y , Wang Y . Spatial transcriptomics in development and disease. Mol Biomed. 2023;4(1):32. 37806992 10.1186/s43556-023-00144-0 PMC10560656

[10] Chen J , Suo S , Tam PP , Han JJ , Peng G , Jing N . Spatial transcriptomic analysis of cryosectioned tissue samples with Geo‐seq. Nat Protoc. 2017;12(3):566‐580. 28207000 10.1038/nprot.2017.003

[11] Boisset JC , Vivie J , Grun D , Muraro MJ , Lyubimova A , van Oudenaarden A . Mapping the physical network of cellular interactions. Nat Methods. 2018;15(7):547‐553. 29786092 10.1038/s41592-018-0009-z

[12] Lovatt D , Ruble BK , Lee J , et al. Transcriptome in vivo analysis (TIVA) of spatially defined single cells in live tissue. Nat Methods. 2014;11(2):190‐196. 24412976 10.1038/nmeth.2804 PMC3964595

[13] Medaglia C , Giladi A , Stoler‐Barak L , et al. Spatial reconstruction of immune niches by combining photoactivatable reporters and scRNA‐seq. Science. 2017;358(6370):1622‐1626. 29217582 10.1126/science.aao4277 PMC7234837

[14] Merritt CR , Ong GT , Church SE , et al. Multiplex digital spatial profiling of proteins and RNA in fixed tissue. Nat Biotechnol. 2020;38(5):586‐599. 32393914 10.1038/s41587-020-0472-9

[15] Kishi JY , Liu N , West ER , et al. Light‐Seq: light‐directed in situ barcoding of biomolecules in fixed cells and tissues for spatially indexed sequencing. Nat Methods. 2022;19(11):1393‐1402. 36216958 10.1038/s41592-022-01604-1 PMC9636025

[16] Junker JP , Noel ES , Guryev V , et al. Genome‐wide RNA Tomography in the zebrafish embryo. Cell. 2014;159(3):662‐675. 25417113 10.1016/j.cell.2014.09.038

[17] Schede HH , Schneider CG , Stergiadou J , et al. Spatial tissue profiling by imaging‐free molecular tomography. Nat Biotechnol. 2021;39(8):968‐977. 33875865 10.1038/s41587-021-00879-7

[18] Femino AM , Fay FS , Fogarty K , Singer RH . Visualization of single RNA transcripts in situ. Science. 1998;280(5363):585‐590. 9554849 10.1126/science.280.5363.585

[19] Larsson C , Grundberg I , Soderberg O , Nilsson M . In situ detection and genotyping of individual mRNA molecules. Nat Methods. 2010;7(5):395‐397. 20383134 10.1038/nmeth.1448

[20] Ke R , Mignardi M , Pacureanu A , et al. In situ sequencing for RNA analysis in preserved tissue and cells. Nat Methods. 2013;10(9):857‐860. 23852452 10.1038/nmeth.2563

[21] Lee JH , Daugharthy ER , Scheiman J , et al. Fluorescent in situ sequencing (FISSEQ) of RNA for gene expression profiling in intact cells and tissues. Nat Protoc. 2015;10(3):442‐458. 25675209 10.1038/nprot.2014.191 PMC4327781

[22] Lee JH , Daugharthy ER , Scheiman J , et al. Highly multiplexed subcellular RNA sequencing in situ. Science. 2014;343(6177):1360‐1363. 24578530 10.1126/science.1250212 PMC4140943

[23] Wang X , Allen WE , Wright MA , et al. Three‐dimensional intact‐tissue sequencing of single‐cell transcriptional states. Science. 2018;361(6400):eaat5691. 29930089 10.1126/science.aat5691 PMC6339868

[24] Chen X , Sun YC , Church GM , Lee JH , Zador AM . Efficient in situ barcode sequencing using padlock probe‐based BaristaSeq. Nucleic Acids Res. 2018;46(4):e22. 29190363 10.1093/nar/gkx1206 PMC5829746

[25] Alon S , Goodwin DR , Sinha A , et al. Expansion sequencing: Spatially precise in situ transcriptomics in intact biological systems. Science. 2021;371(6528):eaax2656. 33509999 10.1126/science.aax2656 PMC7900882

[26] Gyllborg D , Langseth CM , Qian X , et al. Hybridization‐based in situ sequencing (HybISS) for spatially resolved transcriptomics in human and mouse brain tissue. Nucleic Acids Res. 2020;48(19):e112. 32990747 10.1093/nar/gkaa792 PMC7641728

[27] Janesick A , Shelansky R , Gottscho AD , et al. High resolution mapping of the tumor microenvironment using integrated single‐cell, spatial and in situ analysis. Nat Commun. 2023;14(1):8353. 38114474 10.1038/s41467-023-43458-x PMC10730913

[28] Seo ES , Lee B , Hwang I , Kim J‐Y , Park K , Park W‐Y . Decoding spatial organization maps and context‐specific landscapes of breast cancer and its microenvironment via high‐resolution spatial transcriptomic analysis. bioRxiv. 2023:2023.2010.2025.563904.

[29] Haga Y , Sakamoto Y , Kajiya K , et al. Whole‐genome sequencing reveals the molecular implications of the stepwise progression of lung adenocarcinoma. Nat Commun. 2023;14(1):8375. 38102134 10.1038/s41467-023-43732-y PMC10724178

[30] Wang F , Flanagan J , Su N , et al. RNAscope: a novel in situ RNA analysis platform for formalin‐fixed, paraffin‐embedded tissues. J Mol Diagn. 2012;14(1):22‐29. 22166544 10.1016/j.jmoldx.2011.08.002 PMC3338343

[31] Shah S , Lubeck E , Schwarzkopf M , et al. Single‐molecule RNA detection at depth by hybridization chain reaction and tissue hydrogel embedding and clearing. Development. 2016;143(15):2862‐2867. 27342713 10.1242/dev.138560 PMC5004914

[32] Codeluppi S , Borm LE , Zeisel A , et al. Spatial organization of the somatosensory cortex revealed by osmFISH. Nat Methods. 2018;15(11):932‐935. 30377364 10.1038/s41592-018-0175-z

[33] Lubeck E , Coskun AF , Zhiyentayev T , Ahmad M , Cai L . Single‐cell in situ RNA profiling by sequential hybridization. Nat Methods. 2014;11(4):360‐361. 24681720 10.1038/nmeth.2892 PMC4085791

[34] Eng CL , Lawson M , Zhu Q , et al. Transcriptome‐scale super‐resolved imaging in tissues by RNA seqFISH. Nature. 2019;568(7751):235‐239. 30911168 10.1038/s41586-019-1049-y PMC6544023

[35] Chen KH , Boettiger AN , Moffitt JR , Wang S , Zhuang X . RNA imaging. Spatially resolved, highly multiplexed RNA profiling in single cells. Science. 2015;348(6233):aaa6090. 25858977 10.1126/science.aaa6090 PMC4662681

[36] Xia C , Fan J , Emanuel G , Hao J , Zhuang X . Spatial transcriptome profiling by MERFISH reveals subcellular RNA compartmentalization and cell cycle‐dependent gene expression. Proc Natl Acad Sci USA. 2019;116(39):19490‐19499. 31501331 10.1073/pnas.1912459116 PMC6765259

[37] Chiba K , Lorbeer FK , Shain AH , et al. Mutations in the promoter of the telomerase gene TERT contribute to tumorigenesis by a two‐step mechanism. Science. 2017;357(6358):1416‐1420. 28818973 10.1126/science.aao0535 PMC5942222

[38] He S , Bhatt R , Brown C , et al. High‐plex imaging of RNA and proteins at subcellular resolution in fixed tissue by spatial molecular imaging. Nat Biotechnol. 2022;40(12):1794‐1806. 36203011 10.1038/s41587-022-01483-z

[39] Groiss S , Pabst D , Faber C , et al. Highly resolved spatial transcriptomics for detection of rare events in cells. bioRxiv. 2021:2021.2010.2011.463936.

[40] Stahl PL , Salmen F , Vickovic S , et al. Visualization and analysis of gene expression in tissue sections by spatial transcriptomics. Science. 2016;353(6294):78‐82. 27365449 10.1126/science.aaf2403

[41] Cheng M , Jiang Y , Xu J , et al. Spatially resolved transcriptomics: a comprehensive review of their technological advances, applications, and challenges. J Genet Genomics. 2023;50(9):625‐640. 36990426 10.1016/j.jgg.2023.03.011

[42] Rodriques SG , Stickels RR , Goeva A , et al. Slide‐seq: A scalable technology for measuring genome‐wide expression at high spatial resolution. Science. 2019;363(6434):1463‐1467. 30923225 10.1126/science.aaw1219 PMC6927209

[43] Vickovic S , Eraslan G , Salmen F , et al. High‐definition spatial transcriptomics for in situ tissue profiling. Nat Methods. 2019;16(10):987‐990. 31501547 10.1038/s41592-019-0548-y PMC6765407

[44] Srivatsan SR , Regier MC , Barkan E , et al. Embryo‐scale, single‐cell spatial transcriptomics. Science. 2021;373(6550):111‐117. 34210887 10.1126/science.abb9536 PMC9118175

[45] Cho CS , Xi J , Si Y , et al. Microscopic examination of spatial transcriptome using Seq‐Scope. Cell. 2021;184(13):3559‐3572 e3522. 34115981 10.1016/j.cell.2021.05.010 PMC8238917

[46] Liu Y , Yang M , Deng Y , et al. High‐spatial‐resolution multi‐omics sequencing via deterministic barcoding in tissue. Cell. 2020;183(6):1665‐1681 e1618. 33188776 10.1016/j.cell.2020.10.026 PMC7736559

[47] Chen A , Liao S , Cheng M , et al. Spatiotemporal transcriptomic atlas of mouse organogenesis using DNA nanoball‐patterned arrays. Cell. 2022;185(10):1777‐1792 e1721. 35512705 10.1016/j.cell.2022.04.003

[48] Duan H , Cheng T , Cheng H . Spatially resolved transcriptomics: advances and applications. Blood Sci. 2023;5(1):1‐14. 36742187 10.1097/BS9.0000000000000141 PMC9891446

[49] Moses L , Pachter L . Museum of spatial transcriptomics. Nat Methods. 2022;19(5):534‐546. 35273392 10.1038/s41592-022-01409-2

[50] Seferbekova Z , Lomakin A , Yates LR , Gerstung M . Spatial biology of cancer evolution. Nat Rev Genet. 2023;24(5):295‐313. 36494509 10.1038/s41576-022-00553-x

[51] Wu Y , Cheng Y , Wang X , Fan J , Gao Q . Spatial omics: Navigating to the golden era of cancer research. Clin Transl Med. 2022;12(1):e696. 35040595 10.1002/ctm2.696 PMC8764875

[52] Stickels RR , Murray E , Kumar P , et al. Highly sensitive spatial transcriptomics at near‐cellular resolution with Slide‐seqV2. Nat Biotechnol. 2021;39(3):313‐319. 33288904 10.1038/s41587-020-0739-1 PMC8606189

[53] Cao J , Zheng Z , Sun D , et al. Decoder‐seq enhances mRNA capture efficiency in spatial RNA sequencing. Nat Biotechnol. 2024. 10.1038/s41587-023-02086-y 38228777

[54] Song X , Guo P , Xia K , et al. Spatial transcriptomics reveals light‐induced chlorenchyma cells involved in promoting shoot regeneration in tomato callus. Proc Natl Acad Sci USA. 2023;120(38):e2310163120. 37703282 10.1073/pnas.2310163120 PMC10515167

[55] Liao J , Lu X , Shao X , Zhu L , Fan X . Uncovering an organ's molecular architecture at single‐cell resolution by spatially resolved transcriptomics. Trends Biotechnol. 2021;39(1):43‐58. 32505359 10.1016/j.tibtech.2020.05.006

[56] You Y , Fu Y , Li L , et al. Systematic comparison of sequencing‐based spatial transcriptomic methods. Nat Methods. 2024;21(9):1743‐1754. 38965443 10.1038/s41592-024-02325-3 PMC11399101

[57] Emmert‐Buck MR , Bonner RF , Smith PD , et al. Laser capture microdissection. Science. 1996;274(5289):998‐1001. 8875945 10.1126/science.274.5289.998

[58] Zhou Q , Xu X , Li M , et al. Laser capture microdissection transcriptome (LCM RNA‐seq) reveals BcDFR is a key gene in anthocyanin synthesis of non‐heading Chinese cabbage. BMC Genomics. 2024;25(1):425. 38684983 10.1186/s12864-024-10341-y PMC11059580

[59] Oliveira MF , Romero JP , Chung M , et al. Characterization of immune cell populations in the tumor microenvironment of colorectal cancer using high definition spatial profiling. bioRxiv. 2024;2024.2006.2004.597233.

[60] Rademacher A , Huseynov A , Bortolomeazzi M , et al. Comparison of spatial transcriptomics technologies using tumor cryosections. bioRxiv. 2024;2024.2004.2003.586404. 10.1186/s13059-025-03624-4 PMC12180266 40542418

[61] Wang H , Huang R , Nelson J , et al. Systematic benchmarking of imaging spatial transcriptomics platforms in FFPE tissues. bioRxiv. 2023:2023.2012.2007.570603. 10.1038/s41467-025-64990-y PMC12635344 41266321

[62] Hartman A , Satija R . Comparative analysis of multiplexed in situ gene expression profiling technologies. bioRxiv. 2024.

[63] Zeng Z , Li Y , Li Y , Luo Y . Statistical and machine learning methods for spatially resolved transcriptomics data analysis. Genome Biol. 2022;23(1):83. 35337374 10.1186/s13059-022-02653-7 PMC8951701

[64] Hu J , Schroeder A , Coleman K , Chen C , Auerbach BJ , Li M . Statistical and machine learning methods for spatially resolved transcriptomics with histology. Comput Struct Biotechnol J. 2021;19:3829‐3841. 34285782 10.1016/j.csbj.2021.06.052 PMC8273359

[65] Fang S , Chen B , Zhang Y , et al. Computational approaches and challenges in spatial transcriptomics. Genomics Proteomics Bioinformatics. 2023;21(1):24‐47. 36252814 10.1016/j.gpb.2022.10.001 PMC10372921

[66] Schurch CM , Bhate SS , Barlow GL , et al. Coordinated cellular neighborhoods orchestrate antitumoral immunity at the colorectal cancer invasive front. Cell. 2020;182(5):1341‐1359 e1319. 32763154 10.1016/j.cell.2020.07.005 PMC7479520

[67] Elyanow R , Zeira R , Land M , Raphael BJ . STARCH: copy number and clone inference from spatial transcriptomics data. Phys Biol. 2021;18(3):035001. 33022659 10.1088/1478-3975/abbe99 PMC9876615

[68] Bhate SS , Barlow GL , Schurch CM , Nolan GP . Tissue schematics map the specialization of immune tissue motifs and their appropriation by tumors. Cell Syst. 2022;13(2):109‐130 e106. 34653369 10.1016/j.cels.2021.09.012 PMC12588373

[69] Palla G , Fischer DS , Regev A , Theis FJ . Spatial components of molecular tissue biology. Nat Biotechnol. 2022;40(3):308‐318. 35132261 10.1038/s41587-021-01182-1

[70] Hafemeister C , Satija R . Normalization and variance stabilization of single‐cell RNA‐seq data using regularized negative binomial regression. Genome Biol. 2019;20(1):296. 31870423 10.1186/s13059-019-1874-1 PMC6927181

[71] Stuart T , Butler A , Hoffman P , et al. Comprehensive integration of single‐cell data. Cell. 2019;177(7):1888‐1902 e1821. 31178118 10.1016/j.cell.2019.05.031 PMC6687398

[72] Lun AT , Bach K , Marioni JC . Pooling across cells to normalize single‐cell RNA sequencing data with many zero counts. Genome Biol. 2016;17:75. 27122128 10.1186/s13059-016-0947-7 PMC4848819

[73] Korsunsky I , Millard N , Fan J , et al. Fast, sensitive and accurate integration of single‐cell data with harmony. Nat Methods. 2019;16(12):1289‐1296. 31740819 10.1038/s41592-019-0619-0 PMC6884693

[74] Satija R , Farrell JA , Gennert D , Schier AF , Regev A . Spatial reconstruction of single‐cell gene expression data. Nat Biotechnol. 2015;33(5):495‐502. 25867923 10.1038/nbt.3192 PMC4430369

[75] Wolf FA , Angerer P , Theis FJ . SCANPY: large‐scale single‐cell gene expression data analysis. Genome Biol. 2018;19(1):15. 29409532 10.1186/s13059-017-1382-0 PMC5802054

[76] Gayoso A , Lopez R , Xing G , et al. A Python library for probabilistic analysis of single‐cell omics data. Nat Biotechnol. 2022;40(2):163‐166. 35132262 10.1038/s41587-021-01206-w

[77] Edsgard D , Johnsson P , Sandberg R . Identification of spatial expression trends in single‐cell gene expression data. Nat Methods. 2018;15(5):339‐342. 29553578 10.1038/nmeth.4634 PMC6314435

[78] Wu Y , Hu Q , Wang S , et al. Highly Regional Genes: graph‐based gene selection for single‐cell RNA‐seq data. J Genet Genomics. 2022;49(9):891‐899. 35144027 10.1016/j.jgg.2022.01.004

[79] Efremova M , Vento‐Tormo M , Teichmann SA , Vento‐Tormo R . CellPhoneDB: inferring cell‐cell communication from combined expression of multi‐subunit ligand‐receptor complexes. Nat Protoc. 2020;15(4):1484‐1506. 32103204 10.1038/s41596-020-0292-x

[80] Jin S , Guerrero‐Juarez CF , Zhang L , et al. Inference and analysis of cell‐cell communication using CellChat. Nat Commun. 2021;12(1):1088. 33597522 10.1038/s41467-021-21246-9 PMC7889871

[81] Bergen V , Lange M , Peidli S , Wolf FA , Theis FJ . Generalizing RNA velocity to transient cell states through dynamical modeling. Nat Biotechnol. 2020;38(12):1408‐1414. 32747759 10.1038/s41587-020-0591-3

[82] Street K , Risso D , Fletcher RB , et al. Slingshot: cell lineage and pseudotime inference for single‐cell transcriptomics. BMC Genomics. 2018;19(1):477. 29914354 10.1186/s12864-018-4772-0 PMC6007078

[83] La Manno G , Soldatov R , Zeisel A , et al. RNA velocity of single cells. Nature. 2018;560(7719):494‐498. 30089906 10.1038/s41586-018-0414-6 PMC6130801

[84] Kleino I , Frolovaite P , Suomi T , Elo LL . Computational solutions for spatial transcriptomics. Comput Struct Biotechnol J. 2022;20:4870‐4884. 36147664 10.1016/j.csbj.2022.08.043 PMC9464853

[85] Stoltzfus CR , Filipek J , Gern BH , et al. CytoMAP: a spatial analysis toolbox reveals features of myeloid cell organization in lymphoid tissues. Cell Rep. 2020;31(3):107523. 32320656 10.1016/j.celrep.2020.107523 PMC7233132

[86] Pham D , Tan X , Xu J , et al. stLearn: integrating spatial location, tissue morphology and gene expression to find cell types, cell‐cell interactions and spatial trajectories within undissociated tissues. bioRxiv. 2020;2020.2005.2031.125658.

[87] Dries R , Zhu Q , Dong R , et al. Giotto: a toolbox for integrative analysis and visualization of spatial expression data. Genome Biol. 2021;22(1):78. 33685491 10.1186/s13059-021-02286-2 PMC7938609

[88] Kueckelhaus J , Ehr Jv , Ravi VM , et al. Inferring spatially transient gene expression pattern from spatial transcriptomic studies. bioRxiv. 2020;2020.2010.2020.346544.

[89] Biancalani T , Scalia G , Buffoni L , et al. Deep learning and alignment of spatially resolved single‐cell transcriptomes with Tangram. Nat Methods. 2021;18(11):1352‐1362. 34711971 10.1038/s41592-021-01264-7 PMC8566243

[90] Bergenstrahle J , Larsson L , Lundeberg J . Seamless integration of image and molecular analysis for spatial transcriptomics workflows. BMC Genomics. 2020;21(1):482. 32664861 10.1186/s12864-020-06832-3 PMC7386244

[91] Sztanka‐Toth TR , Jens M , Karaiskos N , Rajewsky N . Spacemake: processing and analysis of large‐scale spatial transcriptomics data. Gigascience. 2022;11:giac064. 35852420 10.1093/gigascience/giac064 PMC9295369

[92] Dong R , Yuan GC . SpatialDWLS: accurate deconvolution of spatial transcriptomic data. Genome Biol. 2021;22(1):145. 33971932 10.1186/s13059-021-02362-7 PMC8108367

[93] Kleshchevnikov V , Shmatko A , Dann E , et al. Cell2location maps fine‐grained cell types in spatial transcriptomics. Nat Biotechnol. 2022;40(5):661‐671. 35027729 10.1038/s41587-021-01139-4

[94] Zhao E , Stone MR , Ren X , et al. Spatial transcriptomics at subspot resolution with BayesSpace. Nat Biotechnol. 2021;39(11):1375‐1384. 34083791 10.1038/s41587-021-00935-2 PMC8763026

[95] Svensson V , Teichmann SA , Stegle O . SpatialDE: identification of spatially variable genes. Nat Methods. 2018;15(5):343‐346. 29553579 10.1038/nmeth.4636 PMC6350895

[96] Cable DM , Murray E , Zou LS , et al. Robust decomposition of cell type mixtures in spatial transcriptomics. Nat Biotechnol. 2022;40(4):517‐526. 33603203 10.1038/s41587-021-00830-w PMC8606190

[97] Abdelaal T , Mourragui S , Mahfouz A , Reinders MJT . SpaGE: spatial gene enhancement using scRNA‐seq. Nucleic Acids Res. 2020;48(18):e107. 32955565 10.1093/nar/gkaa740 PMC7544237

[98] Liu J , Gao C , Sodicoff J , Kozareva V , Macosko EZ , Welch JD . Jointly defining cell types from multiple single‐cell datasets using LIGER. Nat Protoc. 2020;15(11):3632‐3662. 33046898 10.1038/s41596-020-0391-8 PMC8132955

[99] Cang Z , Nie Q . Inferring spatial and signaling relationships between cells from single cell transcriptomic data. Nat Commun. 2020;11(1):2084. 32350282 10.1038/s41467-020-15968-5 PMC7190659

[100] Abdelaal T , Lelieveldt BPF , Reinders MJT , Mahfouz A . SIRV: Spatial inference of RNA velocity at the single‐cell resolution. bioRxiv. 2021;2021.2007.2026.453774. 10.1093/nargab/lqae100 PMC11302586 39108639

[101] Li B , Zhang W , Guo C , et al. Benchmarking spatial and single‐cell transcriptomics integration methods for transcript distribution prediction and cell type deconvolution. Nat Methods. 2022;19(6):662‐670. 35577954 10.1038/s41592-022-01480-9

[102] Cheng A , Hu G , Li WV . Benchmarking cell‐type clustering methods for spatially resolved transcriptomics data. Brief Bioinform. 2023;24(1):bbac475. 36410733 10.1093/bib/bbac475 PMC9851325

[103] Yuan Z , Zhao F , Lin S , et al. Benchmarking spatial clustering methods with spatially resolved transcriptomics data. Nat Methods. 2024;21(4):712‐722. 38491270 10.1038/s41592-024-02215-8

[104] Yan L , Sun X . Benchmarking and integration of methods for deconvoluting spatial transcriptomic data. Bioinformatics. 2023;39(1):btac805. 36515467 10.1093/bioinformatics/btac805 PMC9825747

[105] Ma Y , Zhou X . Spatially informed cell‐type deconvolution for spatial transcriptomics. Nat Biotechnol. 2022;40(9):1349‐1359. 35501392 10.1038/s41587-022-01273-7 PMC9464662

[106] Miller BF , Huang F , Atta L , Sahoo A , Fan J . Reference‐free cell type deconvolution of multi‐cellular pixel‐resolution spatially resolved transcriptomics data. Nat Commun. 2022;13(1):2339. 35487922 10.1038/s41467-022-30033-z PMC9055051

[107] Yang C , Sin D , Ng R . SMART: reference‐free deconvolution for spatial transcriptomics using marker‐gene‐assisted topic models. bioRxiv. 2023;2023.2006.2020.545793.

[108] Shen R , Liu L , Wu Z , et al. Spatial‐ID: a cell typing method for spatially resolved transcriptomics via transfer learning and spatial embedding. Nat Commun. 2022;13(1):7640. 36496406 10.1038/s41467-022-35288-0 PMC9741613

[109] Garrido‐Trigo A , Corraliza AM , Veny M , et al. Macrophage and neutrophil heterogeneity at single‐cell spatial resolution in human inflammatory bowel disease. Nat Commun. 2023;14(1):4506. 37495570 10.1038/s41467-023-40156-6 PMC10372067

[110] Littman R , Hemminger Z , Foreman R , et al. Joint cell segmentation and cell type annotation for spatial transcriptomics. Mol Syst Biol. 2021;17(6):e10108. 34057817 10.15252/msb.202010108 PMC8166214

[111] Wang N , Song Y , Hong W , et al. Spatial single‐cell transcriptomic analysis in breast cancer reveals potential biomarkers for PD‐1 blockade therapy. In: Research Square, 2024.

[112] Sun S , Zhu J , Zhou X . Statistical analysis of spatial expression patterns for spatially resolved transcriptomic studies. Nat Methods. 2020;17(2):193‐200. 31988518 10.1038/s41592-019-0701-7 PMC7233129

[113] Andersson A , Lundeberg J . sepal: identifying transcript profiles with spatial patterns by diffusion‐based modeling. Bioinformatics. 2021;37(17):2644‐2650. 33704427 10.1093/bioinformatics/btab164 PMC8428601

[114] Hu J , Li X , Coleman K , et al. SpaGCN: Integrating gene expression, spatial location and histology to identify spatial domains and spatially variable genes by graph convolutional network. Nat Methods. 2021;18(11):1342‐1351. 34711970 10.1038/s41592-021-01255-8

[115] Li K , Yan C , Li C , et al. Computational elucidation of spatial gene expression variation from spatially resolved transcriptomics data. Mol Ther Nucleic Acids. 2022;27:404‐411. 35036053 10.1016/j.omtn.2021.12.009 PMC8728308

[116] Chen C , Kim HJ , Yang P . Evaluating spatially variable gene detection methods for spatial transcriptomics data. Genome Biol. 2024;25(1):18. 38225676 10.1186/s13059-023-03145-y PMC10789051

[117] Lopez R , Nazaret A , Langevin M , et al. A joint model of unpaired data from scRNA‐seq and spatial transcriptomics for imputing missing gene expression measurements. ArXiv. 2019;abs/1905.02269.

[118] Zhang D , Schroeder A , Yan H , et al. Inferring super‐resolution tissue architecture by integrating spatial transcriptomics with histology. Nat Biotechnol. 2024. 10.1038/s41587-023-02019-9 PMC11260191 38168986

[119] Duan Z , Riffle D , Li R , Liu J , Min MR , Zhang J . Impeller: a path‐based heterogeneous graph learning method for spatial transcriptomic data imputation. Bioinformatics. 2024;40(6):btae339. 38806165 10.1093/bioinformatics/btae339 PMC11256934

[120] Dimitrov D , Turei D , Garrido‐Rodriguez M , et al. Comparison of methods and resources for cell‐cell communication inference from single‐cell RNA‐Seq data. Nat Commun. 2022;13(1):3224. 35680885 10.1038/s41467-022-30755-0 PMC9184522

[121] Browaeys R , Saelens W , Saeys Y . NicheNet: modeling intercellular communication by linking ligands to target genes. Nat Methods. 2020;17(2):159‐162. 31819264 10.1038/s41592-019-0667-5

[122] Hu Y , Peng T , Gao L , Tan K. CytoTalk: De novo construction of signal transduction networks using single‐cell transcriptomic data. Sci Adv. 2021;7(16):eabf1356. 33853780 10.1126/sciadv.abf1356 PMC8046375

[123] Cang Z , Zhao Y , Almet AA , et al. Screening cell‐cell communication in spatial transcriptomics via collective optimal transport. Nat Methods. 2023;20(2):218‐228. 36690742 10.1038/s41592-022-01728-4 PMC9911355

[124] Arnol D , Schapiro D , Bodenmiller B , Saez‐Rodriguez J , Stegle O . Modeling cell‐cell interactions from spatial molecular data with spatial variance component analysis. Cell Rep. 2019;29(1):202‐211 e206. 31577949 10.1016/j.celrep.2019.08.077 PMC6899515

[125] Yuan Y , Bar‐Joseph Z . GCNG: graph convolutional networks for inferring gene interaction from spatial transcriptomics data. Genome Biol. 2020;21(1):300. 33303016 10.1186/s13059-020-02214-w PMC7726911

[126] Tanevski J , Flores ROR , Gabor A , Schapiro D , Saez‐Rodriguez J . Explainable multiview framework for dissecting spatial relationships from highly multiplexed data. Genome Biol. 2022;23(1):97. 35422018 10.1186/s13059-022-02663-5 PMC9011939

[127] Wolf FA , Hamey FK , Plass M , et al. PAGA: graph abstraction reconciles clustering with trajectory inference through a topology preserving map of single cells. Genome Biol. 2019;20(1):59. 30890159 10.1186/s13059-019-1663-x PMC6425583

[128] Saelens W , Cannoodt R , Todorov H , Saeys Y . A comparison of single‐cell trajectory inference methods. Nat Biotechnol. 2019;37(5):547‐554. 30936559 10.1038/s41587-019-0071-9

[129] Ruf B , Bruhns M , Babaei S , et al. Tumor‐associated macrophages trigger MAIT cell dysfunction at the HCC invasive margin. Cell. 2023;186(17):3686‐3705 e3632. 37595566 10.1016/j.cell.2023.07.026 PMC10461130

[130] Hu Y , Rong J , Xu Y , et al. Unsupervised and supervised discovery of tissue cellular neighborhoods from cell phenotypes. Nat Methods. 2024;21(2):267‐278. 38191930 10.1038/s41592-023-02124-2 PMC10864185

[131] Shiao SL , Gouin KH 3rd , Ing N , et al. Single‐cell and spatial profiling identify three response trajectories to pembrolizumab and radiation therapy in triple negative breast cancer. Cancer Cell. 2024;42(1):70‐84 e78. 38194915 10.1016/j.ccell.2023.12.012

[132] Wang N , Li X , Ding Z . High‐plex spatial profiling of RNA and protein using digital spatial profiler. Methods Mol Biol. 2023;2660:69‐83. 37191791 10.1007/978-1-0716-3163-8_6

[133] Zhang Q , Abdo R , Iosef C , et al. The spatial transcriptomic landscape of non‐small cell lung cancer brain metastasis. Nat Commun. 2022;13(1):5983. 36216799 10.1038/s41467-022-33365-y PMC9551067

[134] Ling Y , Zhong J , Weng Z , et al. The prognostic value and molecular properties of tertiary lymphoid structures in oesophageal squamous cell carcinoma. Clin Transl Med. 2022;12(10):e1074. 36245289 10.1002/ctm2.1074 PMC9574489

[135] Tripodo C , Bertolazzi G , Cancila V , Morello G , Iannitto E . Pseudotemporal ordering of spatial lymphoid tissue microenvironment profiles trails Unclassified DLBCL at the periphery of the follicle. Front Immunol. 2023;14:1207959. 37680642 10.3389/fimmu.2023.1207959 PMC10482233

[136] Guo J , Tang B , Fu J , et al. High‐plex spatial transcriptomic profiling reveals distinct immune components and the HLA class I/DNMT3A/CD8 modulatory axis in mismatch repair‐deficient endometrial cancer. Cell Oncol (Dordr). 2023. 10.1007/s13402-023-00885-8 PMC11090934 37847338

[137] Langfelder P , Horvath S . WGCNA: an R package for weighted correlation network analysis. BMC Bioinformatics. 2008;9:559. 19114008 10.1186/1471-2105-9-559 PMC2631488

[138] Palla G , Spitzer H , Klein M , et al. Squidpy: a scalable framework for spatial omics analysis. Nat Methods. 2022;19(2):171‐178. 35102346 10.1038/s41592-021-01358-2 PMC8828470

[139] Vahid MR , Brown EL , Steen CB , et al. High‐resolution alignment of single‐cell and spatial transcriptomes with CytoSPACE. Nat Biotechnol. 2023;41(11):1543‐1548. 36879008 10.1038/s41587-023-01697-9 PMC10635828

[140] Tan X , Su A , Tran M , Nguyen Q . SpaCell: integrating tissue morphology and spatial gene expression to predict disease cells. Bioinformatics. 2020;36(7):2293‐2294. 31808789 10.1093/bioinformatics/btz914

[141] Bergenstrahle L , He B , Bergenstrahle J , et al. Super‐resolved spatial transcriptomics by deep data fusion. Nat Biotechnol. 2022;40(4):476‐479. 34845373 10.1038/s41587-021-01075-3

[142] Pham D , Tan X , Balderson B , et al. Robust mapping of spatiotemporal trajectories and cell‐cell interactions in healthy and diseased tissues. Nat Commun. 2023;14(1):7739. 38007580 10.1038/s41467-023-43120-6 PMC10676408

[143] Atta L , Clifton K , Anant M , Aihara G , Fan J . Gene count normalization in single‐cell imaging‐based spatially resolved transcriptomics. Genome Biol. 2024;25(1):153. 38867267 10.1186/s13059-024-03303-w PMC11167774

[144] Singhal V , Chou N , Lee J , et al. BANKSY unifies cell typing and tissue domain segmentation for scalable spatial omics data analysis. Nat Genet. 2024;56(3):431‐441. 38413725 10.1038/s41588-024-01664-3 PMC10937399

[145] Li Z , Zhou X . BASS: multi‐scale and multi‐sample analysis enables accurate cell type clustering and spatial domain detection in spatial transcriptomic studies. Genome Biol. 2022;23(1):168. 35927760 10.1186/s13059-022-02734-7 PMC9351148

[146] Cang Z , Ning X , Nie A , Xu M , Zhang J . SCAN‐IT: Domain segmentation of spatial transcriptomics images by graph neural network. BMVC : proceedings of the British Machine Vision Conference British Machine Vision Conference . 2021;32:406. PMC9552951 36227018

[147] Dong K , Zhang S . Deciphering spatial domains from spatially resolved transcriptomics with an adaptive graph attention auto‐encoder. Nat Commun. 2022;13(1):1739. 35365632 10.1038/s41467-022-29439-6 PMC8976049

[148] Long Y , Ang KS , Li M , et al. Spatially informed clustering, integration, and deconvolution of spatial transcriptomics with GraphST. Nat Commun. 2023;14(1):1155. 36859400 10.1038/s41467-023-36796-3 PMC9977836

[149] Fu H , Xu H , Chong K , et al. Unsupervised spatially embedded deep representation of spatial transcriptomics. bioRxiv. 2021;2021.2006.2015.448542. 10.1186/s13073-024-01283-x PMC10790257 38217035

[150] Elosua‐Bayes M , Nieto P , Mereu E , Gut I , Heyn H . SPOTlight: seeded NMF regression to deconvolute spatial transcriptomics spots with single‐cell transcriptomes. Nucleic Acids Res. 2021;49(9):e50. 33544846 10.1093/nar/gkab043 PMC8136778

[151] Wei R , He S , Bai S , et al. Spatial charting of single‐cell transcriptomes in tissues. Nat Biotechnol. 2022;40(8):1190‐1199. 35314812 10.1038/s41587-022-01233-1 PMC9673606

[152] Liao J , Qian J , Fang Y , et al. De novo analysis of bulk RNA‐seq data at spatially resolved single‐cell resolution. Nat Commun. 2022;13(1):6498. 36310179 10.1038/s41467-022-34271-z PMC9618574

[153] Zhu J , Sabatti C . Integrative spatial single‐cell analysis with graph‐based feature learning. bioRxiv. 2020;2020.2008.2012.248971.

[154] Lopez R , Nazaret A , Langevin M , et al. A joint model of unpaired data from scRNA‐seq and spatial transcriptomics for imputing missing gene expression measurements. bioRxiv. 2019;abs/1905.02269.

[155] Haviv D , Remsik J , Gatie M , et al. The covariance environment defines cellular niches for spatial inference. Nat Biotechnol. 2024. 10.1038/s41587-024-02193-4 PMC11445396 38565973

[156] Yang W , Wang P , Xu S , et al. Deciphering cell‐cell communication at single‐cell resolution for spatial transcriptomics with subgraph‐based graph attention network. Nat Commun. 2024;15(1):7101. 39155292 10.1038/s41467-024-51329-2 PMC11330978

[157] Shao X , Li C , Yang H , et al. Knowledge‐graph‐based cell‐cell communication inference for spatially resolved transcriptomic data with SpaTalk. Nat Commun. 2022;13(1):4429. 35908020 10.1038/s41467-022-32111-8 PMC9338929

[158] Qiu X , Mao Q , Tang Y , et al. Reversed graph embedding resolves complex single‐cell trajectories. Nat Methods. 2017;14(10):979‐982. 28825705 10.1038/nmeth.4402 PMC5764547

[159] Abdelaal T , Lelieveldt BPF , Reinders MJT , Mahfouz A . SIRV: Spatial inference of RNA velocity at the single‐cell resolution. bioRxiv. 2021:2021.2007.2026.453774. 10.1093/nargab/lqae100 PMC11302586 39108639

[160] Sheridan C . Can single‐cell biology realize the promise of precision medicine? Nat Biotechnol. 2024;42(2):159‐162. 38361071 10.1038/s41587-024-02138-x

[161] Wang Q , Zhi Y , Zi M , et al. Spatially resolved transcriptomics technology facilitates cancer research. Adv Sci (Weinh). 2023;10(30):e2302558. 37632718 10.1002/advs.202302558 PMC10602551

[162] Coulson A . How is spatial transcriptomics influencing cancer research and diagnostics? Biotechniques. 2022;73(5):215‐217. 36398881 10.2144/btn-2022-0111

[163] Erfanian N , Nasseri S , Miraki Feriz A , Safarpour H , Namaei MH . Characterization of Wnt signaling pathway under treatment of Lactobacillus acidophilus postbiotic in colorectal cancer using an integrated in silico and in vitro analysis. Sci Rep. 2023;13(1):22988. 38151510 10.1038/s41598-023-50047-x PMC10752892

[164] Ferri‐Borgogno S , Burks JK , Seeley EH , et al. Molecular, metabolic, and subcellular mapping of the tumor immune microenvironment via 3D targeted and non‐targeted multiplex multi‐omics analyses. Cancers (Basel). 2024;16(5):846. 38473208 10.3390/cancers16050846 PMC10930466

[165] Yeh CY , Aguirre K , Laveroni O , et al. Mapping ovarian cancer spatial organization uncovers immune evasion drivers at the genetic, cellular, and tissue level. bioRxiv. 2023;2023.2010.2016.562592.

[166] Magen A , Hamon P , Fiaschi N , et al. Intratumoral dendritic cell‐CD4(+) T helper cell niches enable CD8(+) T cell differentiation following PD‐1 blockade in hepatocellular carcinoma. Nat Med. 2023;29(6):1389‐1399. 37322116 10.1038/s41591-023-02345-0 PMC11027932

[167] Chen JH , Nieman LT , Spurrell M , et al. Human lung cancer harbors spatially organized stem‐immunity hubs associated with response to immunotherapy. Nat Immunol. 2024;25(4):644‐658. 38503922 10.1038/s41590-024-01792-2 PMC12096941

[168] Hara T , Chanoch‐Myers R , Mathewson ND , et al. Interactions between cancer cells and immune cells drive transitions to mesenchymal‐like states in glioblastoma. Cancer Cell. 2021;39(6):779‐792 e711. 34087162 10.1016/j.ccell.2021.05.002 PMC8366750

[169] Li Z , Pai R , Gupta S , et al. Presence of onco‐fetal neighborhoods in hepatocellular carcinoma is associated with relapse and response to immunotherapy. Nat Cancer. 2024;5(1):167‐186. 38168935 10.1038/s43018-023-00672-2

[170] Chen C , Guo Q , Liu Y , et al. Single‐cell and spatial transcriptomics reveal POSTN(+) cancer‐associated fibroblasts correlated with immune suppression and tumour progression in non‐small cell lung cancer. Clin Transl Med. 2023;13(12):e1515. 38115703 10.1002/ctm2.1515 PMC10731139

[171] Qin P , Chen H , Wang Y , et al. Cancer‐associated fibroblasts undergoing neoadjuvant chemotherapy suppress rectal cancer revealed by single‐cell and spatial transcriptomics. Cell Rep Med. 2023;4(10):101231. 37852187 10.1016/j.xcrm.2023.101231 PMC10591051

[172] Wu L , Yan J , Bai Y , et al. An invasive zone in human liver cancer identified by Stereo‐seq promotes hepatocyte‐tumor cell crosstalk, local immunosuppression and tumor progression. Cell Res. 2023;33(8):585‐603. 37337030 10.1038/s41422-023-00831-1 PMC10397313

[173] Ou Z , Lin S , Qiu J , et al. Single‐Nucleus RNA Sequencing and Spatial Transcriptomics Reveal the Immunological Microenvironment of Cervical Squamous Cell Carcinoma. Adv Sci (Weinh). 2022;9(29):e2203040. 35986392 10.1002/advs.202203040 PMC9561780

[174] Karras P , Bordeu I , Pozniak J , et al. A cellular hierarchy in melanoma uncouples growth and metastasis. Nature. 2022;610(7930):190‐198. 36131018 10.1038/s41586-022-05242-7 PMC10439739

[175] Zhang R , Feng Y , Ma W , et al. Spatial transcriptome unveils a discontinuous inflammatory pattern in proficient mismatch repair colorectal adenocarcinoma. Fundam Res. 2023;3(4):640‐646. 38933545 10.1016/j.fmre.2022.01.036 PMC11197706

[176] Denisenko E , de Kock L , Tan A , et al. Spatial transcriptomics reveals discrete tumour microenvironments and autocrine loops within ovarian cancer subclones. Nat Commun. 2024;15(1):2860. 38570491 10.1038/s41467-024-47271-y PMC10991508

[177] Moffet JJD , Fatunla OE , Freytag L , et al. Spatial architecture of high‐grade glioma reveals tumor heterogeneity within distinct domains. Neurooncol Adv. 2023;5(1):vdad142. 38077210 10.1093/noajnl/vdad142 PMC10699851

[178] Derry JMJ , Burns C , Frazier JP , et al. Trackable intratumor microdosing and spatial profiling provide early insights into activity of investigational agents in the intact tumor microenvironment. Clin Cancer Res. 2023;29(18):3813‐3825. 37389981 10.1158/1078-0432.CCR-23-0827 PMC10502463

[179] Duchatel RJ , Jackson ER , Parackal SG , et al. PI3K/mTOR is a therapeutically targetable genetic dependency in diffuse intrinsic pontine glioma. J Clin Invest. 2024;134(6):e170329. 38319732 10.1172/JCI170329 PMC10940093

[180] Seo ES , Lee B , Hwang I , Kim J‐Y , Park K , Park W‐Y . Decoding spatial organization maps and context‐specific landscapes of breast cancer and its microenvironment via high‐resolution spatial transcriptomic analysis. bioRxiv. 2023;2023.2010.2025.563904.

[181] Mangoli A , Wu S , Liu HQ , et al. Ataxia‐telangiectasia mutated (Atm) disruption sensitizes spatially‐directed H3.3K27M/TP53 diffuse midline gliomas to radiation therapy. bioRxiv. 2023;2023.2010.2018.562892.

[182] Patel AG , Ashenberg O , Collins NB , et al. A spatial cell atlas of neuroblastoma reveals developmental, epigenetic and spatial axis of tumor heterogeneity. bioRxiv. 2024;2024.2001.2007.574538.

[183] Hirz T , Mei S , Sarkar H , et al. Dissecting the immune suppressive human prostate tumor microenvironment via integrated single‐cell and spatial transcriptomic analyses. Nat Commun. 2023;14(1):663. 36750562 10.1038/s41467-023-36325-2 PMC9905093

[184] Andersson A , Larsson L , Stenbeck L , et al. Spatial deconvolution of HER2‐positive breast cancer delineates tumor‐associated cell type interactions. Nat Commun. 2021;12(1):6012. 34650042 10.1038/s41467-021-26271-2 PMC8516894

[185] Mao X , Zhou D , Lin K , et al. Single‐cell and spatial transcriptome analyses revealed cell heterogeneity and immune environment alternations in metastatic axillary lymph nodes in breast cancer. Cancer Immunol Immunother. 2023;72(3):679‐695. 36040519 10.1007/s00262-022-03278-2 PMC10991914

[186] Sun H , Li Y , Zhang Y , et al. The relevance between hypoxia‐dependent spatial transcriptomics and the prognosis and efficacy of immunotherapy in claudin‐low breast cancer. Front Immunol. 2022;13:1042835. 36685583 10.3389/fimmu.2022.1042835 PMC9846556

[187] Nejman D , Livyatan I , Fuks G , et al. The human tumor microbiome is composed of tumor type‐specific intracellular bacteria. Science. 2020;368(6494):973‐980. 32467386 10.1126/science.aay9189 PMC7757858

[188] Galeano Nino JL, Wu H , LaCourse KD , et al. Effect of the intratumoral microbiota on spatial and cellular heterogeneity in cancer. Nature. 2022;611(7937):810‐817. 36385528 10.1038/s41586-022-05435-0 PMC9684076

[189] Wong‐Rolle A , Dong Q , Zhu Y , et al. Spatial meta‐transcriptomics reveal associations of intratumor bacteria burden with lung cancer cells showing a distinct oncogenic signature. J Immunother Cancer. 2022;10(7):e004698. 35793869 10.1136/jitc-2022-004698 PMC9260850

[190] Larroquette M , Guegan JP , Besse B , et al. Spatial transcriptomics of macrophage infiltration in non‐small cell lung cancer reveals determinants of sensitivity and resistance to anti‐PD1/PD‐L1 antibodies. J Immunother Cancer. 2022;10(5):e003890. 35618288 10.1136/jitc-2021-003890 PMC9125754

[191] Zhu J , Fan Y , Xiong Y , et al. Delineating the dynamic evolution from preneoplasia to invasive lung adenocarcinoma by integrating single‐cell RNA sequencing and spatial transcriptomics. Exp Mol Med. 2022;54(11):2060‐2076. 36434043 10.1038/s12276-022-00896-9 PMC9722784

[192] Wang Y , Liu B , Min Q , et al. Spatial transcriptomics delineates molecular features and cellular plasticity in lung adenocarcinoma progression. Cell Discov. 2023;9(1):96. 37723144 10.1038/s41421-023-00591-7 PMC10507052

[193] Nirmal AJ , Maliga Z , Vallius T , et al. The spatial landscape of progression and immunoediting in primary melanoma at single‐cell resolution. Cancer Discov. 2022;12(6):1518‐1541. 35404441 10.1158/2159-8290.CD-21-1357 PMC9167783

[194] Liu Y , Xun Z , Ma K , et al. Identification of a tumour immune barrier in the HCC microenvironment that determines the efficacy of immunotherapy. J Hepatol. 2023;78(4):770‐782. 36708811 10.1016/j.jhep.2023.01.011

[195] Sun Y , Wu P , Zhang Z , et al. Integrated multi‐omics profiling to dissect the spatiotemporal evolution of metastatic hepatocellular carcinoma. Cancer Cell. 2024;42(1):135‐156 e117. 38101410 10.1016/j.ccell.2023.11.010

[196] Hwang WL , Jagadeesh KA , Guo JA , et al. Single‐nucleus and spatial transcriptome profiling of pancreatic cancer identifies multicellular dynamics associated with neoadjuvant treatment. Nat Genet. 2022;54(8):1178‐1191. 35902743 10.1038/s41588-022-01134-8 PMC10290535

[197] Cui Zhou D , Jayasinghe RG , Chen S , et al. Spatially restricted drivers and transitional cell populations cooperate with the microenvironment in untreated and chemo‐resistant pancreatic cancer. Nat Genet. 2022;54(9):1390‐1405. 35995947 10.1038/s41588-022-01157-1 PMC9470535

[198] Hong WF , Zhang F , Wang N , et al. Dynamic immunoediting by macrophages in homologous recombination deficiency‐stratified pancreatic ductal adenocarcinoma. Drug Resist Updat. 2024;76:101115. 39002266 10.1016/j.drup.2024.101115

[199] Wang N , Wang R , Li X , et al. Tumor microenvironment profiles reveal distinct therapy‐oriented proteogenomic characteristics in colorectal cancer. Front Bioeng Biotechnol. 2021;9:757378. 34778231 10.3389/fbioe.2021.757378 PMC8581216

[200] Qi J , Sun H , Zhang Y , et al. Single‐cell and spatial analysis reveal interaction of FAP(+) fibroblasts and SPP1(+) macrophages in colorectal cancer. Nat Commun. 2022;13(1):1742. 35365629 10.1038/s41467-022-29366-6 PMC8976074

[201] Kumar V , Ramnarayanan K , Sundar R , et al. Single‐cell atlas of lineage states, tumor microenvironment, and subtype‐specific expression programs in gastric cancer. Cancer Discov. 2022;12(3):670‐691. 34642171 10.1158/2159-8290.CD-21-0683 PMC9394383

[202] Yeh CY , Aguirre K , Laveroni O , et al. Mapping ovarian cancer spatial organization uncovers immune evasion drivers at the genetic, cellular, and tissue level. bioRxiv. 2023;2023.2010.2016.562592.

[203] Liu X , Zhao S , Wang K , et al. Spatial transcriptomics analysis of esophageal squamous precancerous lesions and their progression to esophageal cancer. Nat Commun. 2023;14(1):4779. 37553345 10.1038/s41467-023-40343-5 PMC10409784

[204] Zheng H , An M , Luo Y , et al. PDGFRalpha(+)ITGA11(+) fibroblasts foster early‐stage cancer lymphovascular invasion and lymphatic metastasis via ITGA11‐SELE interplay. Cancer Cell. 2024;42(4):682‐700 e612. 38428409 10.1016/j.ccell.2024.02.002

[205] Vo T , Balderson B , Jones K , et al. Spatial transcriptomic analysis of Sonic hedgehog medulloblastoma identifies that the loss of heterogeneity and promotion of differentiation underlies the response to CDK4/6 inhibition. Genome Med. 2023;15(1):29. 37127652 10.1186/s13073-023-01185-4 PMC10150495

[206] Ning K , Li Z , Liu H , et al. Perirenal fat thickness significantly associated with prognosis of metastatic renal cell cancer patients receiving anti‐VEGF therapy. Nutrients. 2022;14(16)3388. 36014894 10.3390/nu14163388 PMC9412489

[207] Xie F , Xi N , Han Z , et al. Progress in research on tumor microenvironment‐based spatial omics technologies. Oncol Res. 2023;31(6):877‐885. 37744276 10.32604/or.2023.029494 PMC10513957

[208] Scott EC , Baines AC , Gong Y , et al. Trends in the approval of cancer therapies by the FDA in the twenty‐first century. Nat Rev Drug Discov. 2023;22(8):625‐640. 37344568 10.1038/s41573-023-00723-4

[209] Niu X , Liu W , Zhang Y , et al. Cancer plasticity in therapy resistance: mechanisms and novel strategies. Drug Resist Updat. 2024;76:101114. 38924995 10.1016/j.drup.2024.101114

[210] Zhang L , Chen D , Song D , et al. Clinical and translational values of spatial transcriptomics. Signal Transduct Target Ther. 2022;7(1):111. 35365599 10.1038/s41392-022-00960-w PMC8972902

[211] Wang Y , Mashock M , Tong Z , et al. Changing technologies of RNA sequencing and their applications in clinical oncology. Front Oncol. 2020;10:447. 32328458 10.3389/fonc.2020.00447 PMC7160325

[212] Gan X , Dong W , You W , et al. Spatial multimodal analysis revealed tertiary lymphoid structures as a risk stratification indicator in combined hepatocellular‐cholangiocarcinoma. Cancer Lett. 2024;581:216513. 38036041 10.1016/j.canlet.2023.216513

[213] Kiuru M , Kriner MA , Wong S , et al. High‐plex spatial RNA profiling reveals cell type‒specific biomarker expression during melanoma development. J Invest Dermatol. 2022;142(5):1401‐1412 e1420. 34699906 10.1016/j.jid.2021.06.041 PMC9714472

[214] Ferri‐Borgogno S , Zhu Y , Sheng J , et al. Spatial transcriptomics depict ligand‐receptor cross‐talk heterogeneity at the tumor‐stroma interface in long‐term ovarian cancer survivors. Cancer Res. 2023;83(9):1503‐1516. 36787106 10.1158/0008-5472.CAN-22-1821 PMC10159916

[215] Monkman J , Kim H , Mayer A , et al. Multi‐omic and spatial dissection of immunotherapy response groups in non‐small cell lung cancer. Immunology. 2023;169(4):487‐502. 37022147 10.1111/imm.13646

[216] Park S , Hong C , Cheong J‐H , et al. Abstract 2262: spatial architecture and cellular interactions of tumor immune microenvironment to discover biomarkers and predict immune checkpoint inhibitor response in gastric cancer. Cancer Res. 2023;83(7_Supplement):2262‐2262.37145144

[217] Moutafi M , Martinez‐Morilla S , Garcia‐Milian R , et al. Abstract 2027: Spatial omics and multiplexed imaging to discover new biomarkers of response or resistance to immune checkpoint inhibitors (ICI) in advanced non‐small cell lung cancer (NSCLC). Cancer Res. 2022;82(12_Supplement):2027‐2027.

[218] Yang L , Zhang Z , Dong J , et al. Multi‐dimensional characterization of immunological profiles in small cell lung cancer uncovers clinically relevant immune subtypes with distinct prognoses and therapeutic vulnerabilities. Pharmacol Res. 2023;194:106844. 37392900 10.1016/j.phrs.2023.106844

[219] Zhang Z , Sun X , Liu Y , et al. Spatial transcriptome‐wide profiling of small cell lung cancer reveals intra‐tumoral molecular and subtype heterogeneity. Adv Sci (Weinh). 2024:e2402716. 38896789 10.1002/advs.202402716 PMC11336901

[220] Wang XQ , Danenberg E , Huang CS , et al. Spatial predictors of immunotherapy response in triple‐negative breast cancer. Nature. 2023;621(7980):868‐876. 37674077 10.1038/s41586-023-06498-3 PMC10533410

[221] Cassier P , Eberst L , Garin G , et al. 439O ‐ A first in human, phase I trial of NP137, a first‐in‐class antibody targeting netrin‐1, in patients with advanced refractory solid tumors. Ann Oncol. 2019;30:v159.

[222] Kornauth C , Pemovska T , Vladimer GI , et al. Functional precision medicine provides clinical benefit in advanced aggressive hematologic cancers and identifies exceptional responders. Cancer Discov. 2022;12(2):372‐387. 34635570 10.1158/2159-8290.CD-21-0538 PMC9762339

[223] Doroshow DB , Bhalla S , Beasley MB , et al. PD‐L1 as a biomarker of response to immune‐checkpoint inhibitors. Nat Rev Clin Oncol. 2021;18(6):345‐362. 33580222 10.1038/s41571-021-00473-5

[224] Bruni D , Angell HK , Galon J . The immune contexture and immunoscore in cancer prognosis and therapeutic efficacy. Nat Rev Cancer. 2020;20(11):662‐680. 32753728 10.1038/s41568-020-0285-7

[225] Murciano‐Goroff YR , Warner AB , Wolchok JD . The future of cancer immunotherapy: microenvironment‐targeting combinations. Cell Res. 2020;30(6):507‐519. 32467593 10.1038/s41422-020-0337-2 PMC7264181

[226] Ling AL , Solomon IH , Landivar AM , et al. Clinical trial links oncolytic immunoactivation to survival in glioblastoma. Nature. 2023;623(7985):157‐166. 37853118 10.1038/s41586-023-06623-2 PMC10620094

[227] Yi L , Ning Z , Xu L , et al. The combination treatment of oncolytic adenovirus H101 with nivolumab for refractory advanced hepatocellular carcinoma: an open‐label, single‐arm, pilot study. ESMO Open. 2024;9(2):102239. 38325225 10.1016/j.esmoop.2024.102239 PMC10937204

[228] Jiang M , Li Q , Xu B . Spotlight on ideal target antigens and resistance in antibody‐drug conjugates: strategies for competitive advancement. Drug Resist Updat. 2024;75:101086. 38677200 10.1016/j.drup.2024.101086

[229] Powles T , Valderrama BP , Gupta S , et al. Enfortumab vedotin and pembrolizumab in untreated advanced urothelial cancer. N Engl J Med. 2024;390(10):875‐888. 38446675 10.1056/NEJMoa2312117

[230] Sanmamed MF , Chen L . A paradigm shift in cancer immunotherapy: from enhancement to normalization. Cell. 2018;175(2):313‐326. 30290139 10.1016/j.cell.2018.09.035 PMC6538253

[231] Lu S , Stein JE , Rimm DL , et al. Comparison of biomarker modalities for predicting response to PD‐1/PD‐L1 checkpoint blockade: a systematic review and meta‐analysis. JAMA Oncol. 2019;5(8):1195‐1204. 31318407 10.1001/jamaoncol.2019.1549 PMC6646995

[232] Engblom C , Thrane K , Lin Q , et al. Spatial transcriptomics of B cell and T cell receptors reveals lymphocyte clonal dynamics. Science. 2023;382(6675):eadf8486. 38060664 10.1126/science.adf8486

[233] Schott M , Leon‐Perinan D , Splendiani E , et al. Open‐ST: High‐resolution spatial transcriptomics in 3D. Cell. 2024;187(15):3953‐3972 e3926. 38917789 10.1016/j.cell.2024.05.055

[234] Yang L , Li A , Wang Y , Zhang Y . Intratumoral microbiota: roles in cancer initiation, development and therapeutic efficacy. Signal Transduct Target Ther. 2023;8(1):35. 36646684 10.1038/s41392-022-01304-4 PMC9842669

[235] Lotstedt B , Strazar M , Xavier R , Regev A , Vickovic S . Spatial host‐microbiome sequencing reveals niches in the mouse gut. Nat Biotechnol. 2023;42(9):1394‐1403. 37985876 10.1038/s41587-023-01988-1 PMC11392810

[236] Magoulopoulou A , Qian X , Pediatama Setiabudiawan T , et al. Spatial resolution of mycobacterium tuberculosis bacteria and their surrounding immune environments based on selected key transcripts in mouse lungs. Front Immunol. 2022;13:876321. 35663950 10.3389/fimmu.2022.876321 PMC9157500

